# Supplementary material for: Based on Multi-Activity Integrated Strategy to Screening, Characterization and Quantification of Bioactive Compounds from Red Wine
Source: Molecules. 2021 Nov 8;26(21):6750. doi: 10.3390/molecules26216750 (PMC8587790; doi:10.3390/molecules26216750)
Supplement: Supplementary file 1 [file molecules-26-06750-s001.zip › molecules-1387190-supplementary.pdf]

Supplementary Materials

Article

Based on multi-activity integrated strategy to screening, characterization and quantification of bioactive compounds from red wine

Yuye Gao[1,a], Xie-an Yu[2,a], Bing Wang[2], Guo Yin[2], Jue Wang[2], Tiejie Wang[2,*] and Kaishun Bi[1,*]

[1] School of Pharmacy, Shenyang Pharmaceutical University, Shenyang, Liaoning 110016, China
[2] NMPA Key Laboratory for Quality Research and Evaluation of Traditional Chinese Medicine, Shenzhen Institute for Drug Control, Shenzhen 518057, China
* Correspondence: kaishunbi.syphu@gmail.com
[a] These authors contributed equally.

Table S1. Origin and varietal of the wines.

Samples	Vintage	Vol (%)	Cultivar(s)	Location
S1	2015	13.5	Cabernet Sauvignon	Maipo Vallay, Chile
S2	2018	13	Cabernet Sauvignon	the central valley of Chile
S3	2018	13.5	Cabernet Sauvignon	Maipo Vallay, Chile
S4	2018	13.5	Cabernet Sauvignon	Maipo Vallay and Aconcagua Valley, Chile
S5	2017	13.5	Cabernet Sauvignon	Aconcagua Valley, Chile
S6	2018	13.8	Cabernet Sauvignon	Mendoza, Argentina
S7	2015	14.5	Cabernet Sauvignon	Mendoza, Argentina
S8	2018	15	Cabernet Sauvignon	Mendoza, Argentina
S9	2016	13.3	Cabernet Sauvignon	Mendoza, Argentina
S10	2016	14.1	Cabernet Sauvignon	Mendoza, Argentina
S11	2017	14.5	Cabernet Sauvignon	Clare Valley, Australia
S12	2015	14	Cabernet Sauvignon	Limestone coast, Australia
S13	2018	14.5	Cabernet Sauvignon	South Australia
S14	2017	14	Cabernet Sauvignon	South Australia
S15	2017	14	Cabernet Sauvignon	Clare Valley, Australia
S16	2018	12	Cabernet Sauvignon	Changyu Vineyard, China
S17	2018	14	Cabernet Sauvignon	Ganchengzi District, Qingtongxia, Ningxia, China
S18	2017	14.2	Cabernet Sauvignon	Ningxia Helan Mountain East Foot Production Area, China

S19	2017	13	Cabernet Sauvignon	Changyu Vineyard, China
S20	2019	13	Cabernet Sauvignon	Changyu Vineyard, China
S21	2018	14	Shiraz	Limestone Coast of Australia
S22	2016	14	Shiraz	Great West, Australia
S23	2016	14.5	Shiraz	Hunter Valley, Australia
S24	2016	14	Shiraz	South Australia
S25	2016	14.5	Shiraz	Barossa, Australia
S26	2016	14.5	Shiraz	Pertway, Australia
S27	2016	14.5	Shiraz	Kunawala, Australia
S28	2014	14	Shiraz	Moray Valley, Chile
S29	2016	14	Shiraz	Espinal vineyard, Chile
S30	2018	14.3	Shiraz	Pascual Toso, Argentina
S31	2018	13.5	Merlot	Cachabel Valley, Chile
S32	2018	13.5	Merlot	Curisco Valley, Chile
S33	2017	14.5	Merlot	Konggagua Valley, Chile
S34	2019	13.5	Merlot	Southeast Australia
S35	2014	12.5	Merlot	Southeast Australia

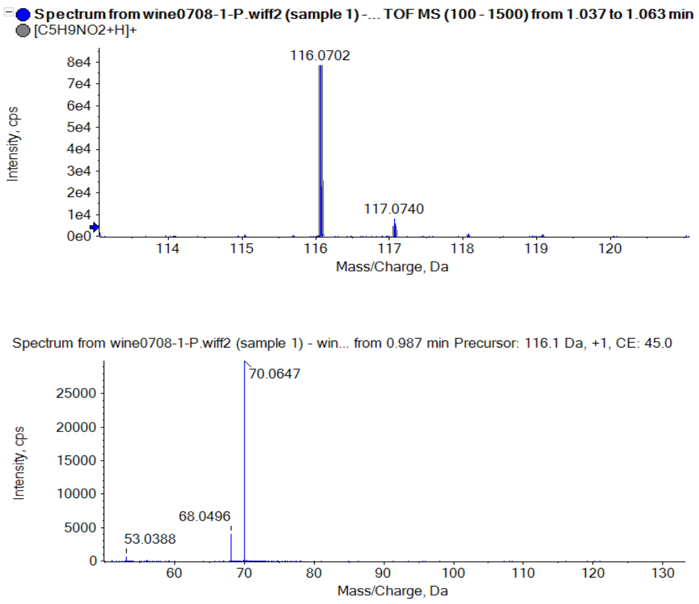

Figure S1. The MS and MS/MS fragments of D-proline.

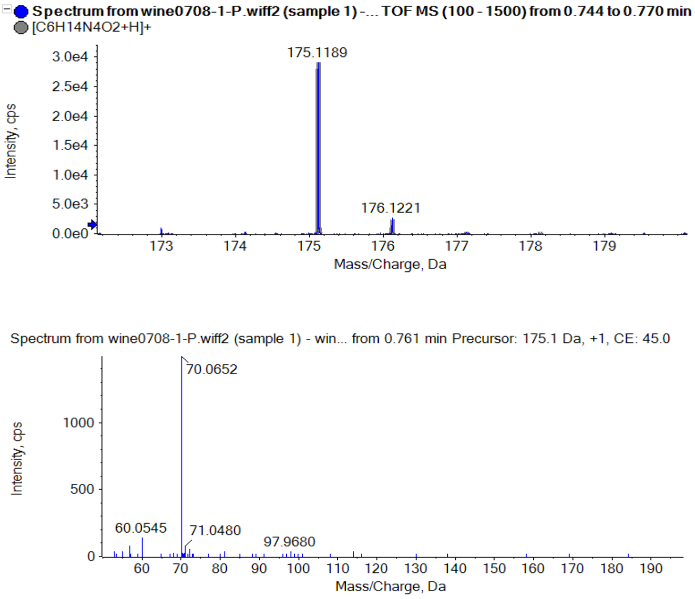

Figure S2. The MS and MS/MS fragments of L-arginine.

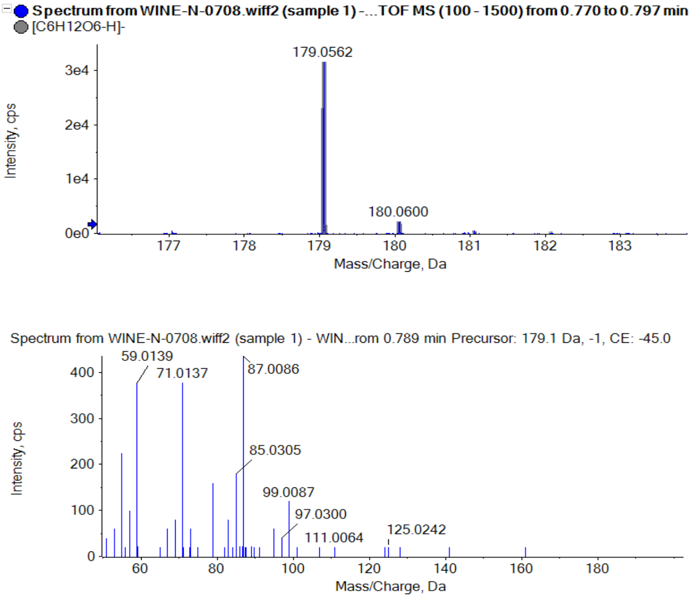

Figure S3. The MS and MS/MS fragments of D-glucose.

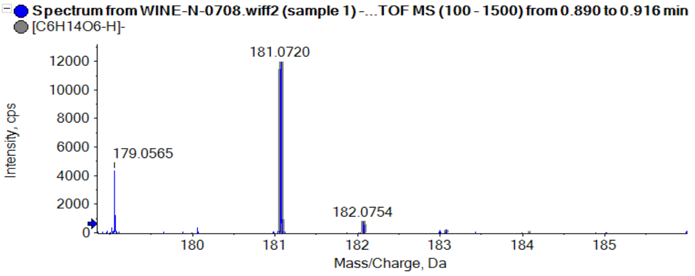

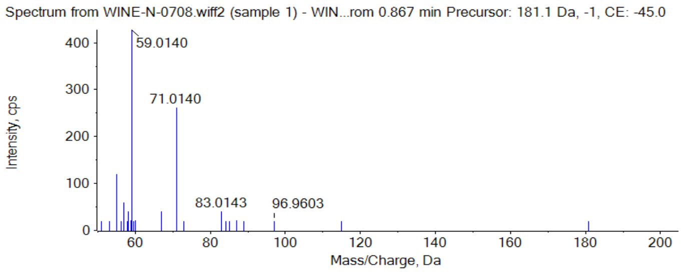

Figure S4. The MS and MS/MS fragments of D-mannitol.

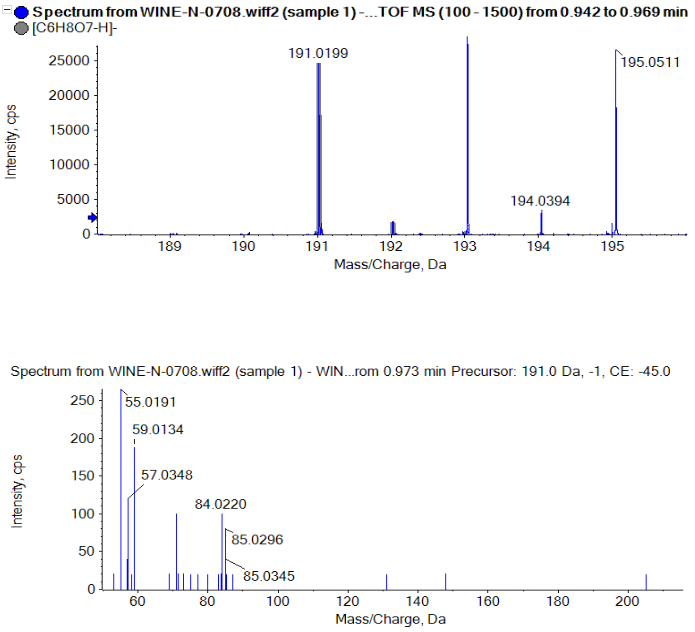

Figure S5. The MS and MS/MS fragments of citric acid.

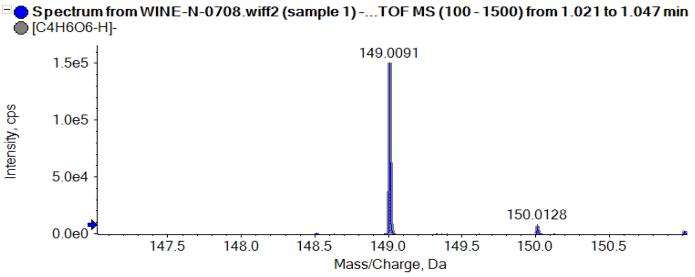

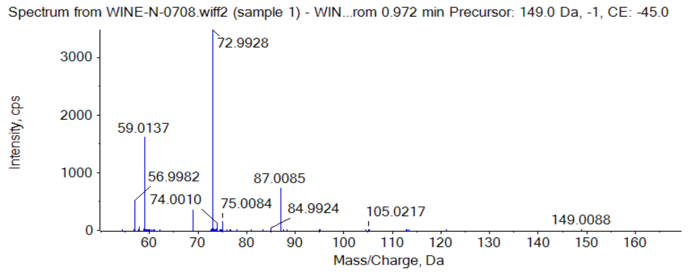

Figure S6. The MS and MS/MS fragments of tartaric acid.

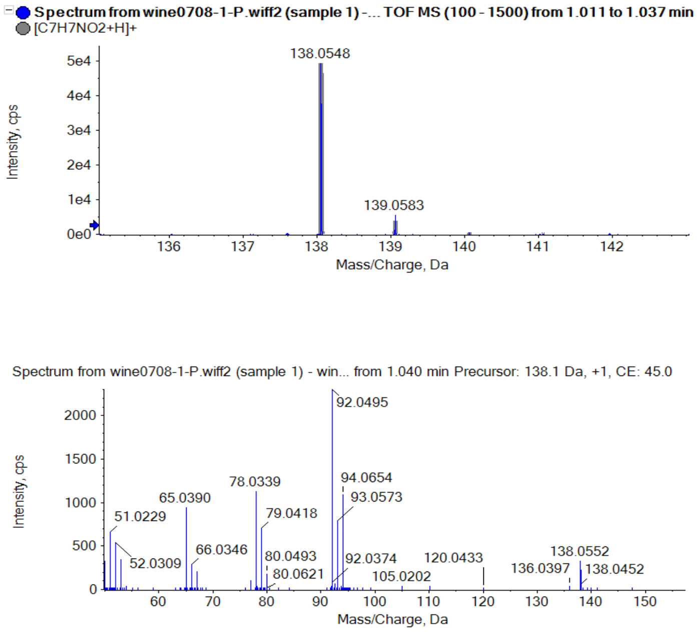

Figure S7. The MS and MS/MS fragments of 4-aminobenzoic acid.

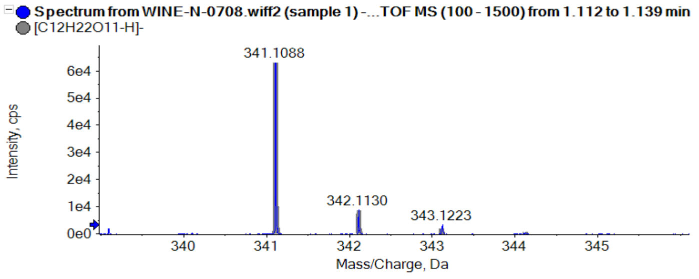

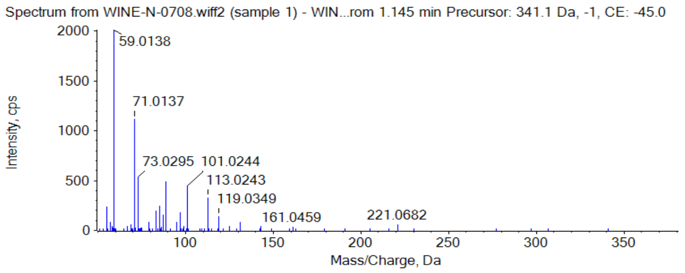

Figure S8. The MS and MS/MS fragments of trehalose.

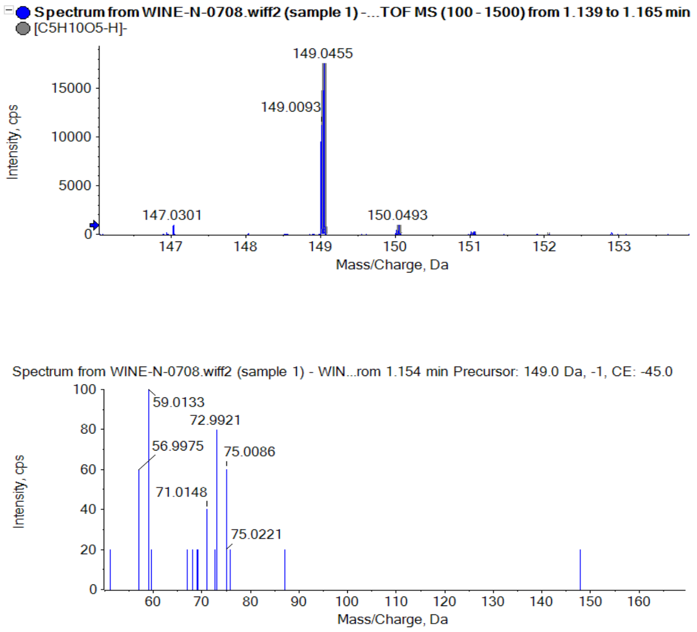

Figure S9. The MS and MS/MS fragments of D-ribose.

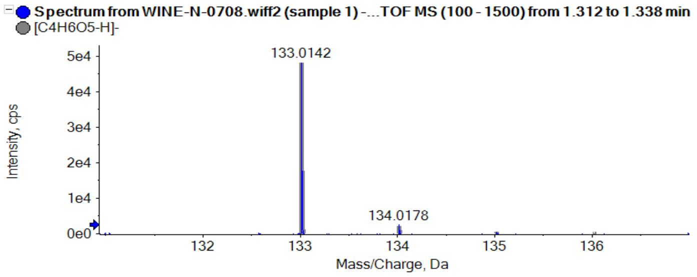

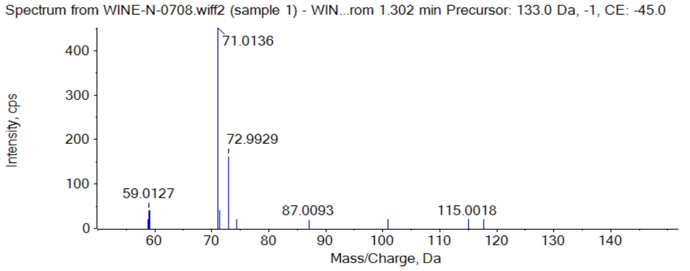

Figure S10. The MS and MS/MS fragments of L-malic acid.

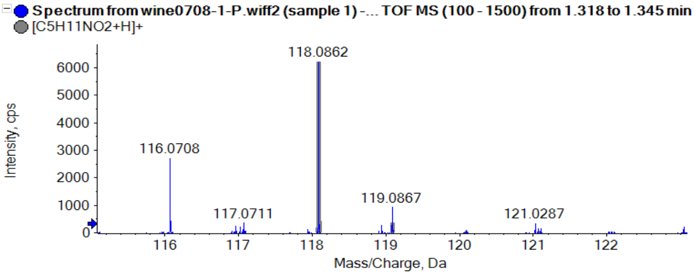

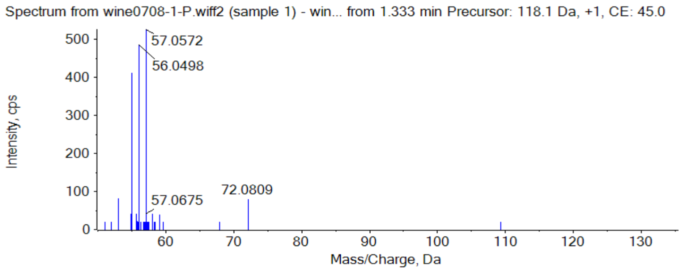

Figure S11. The MS and MS/MS fragments of L-valine.

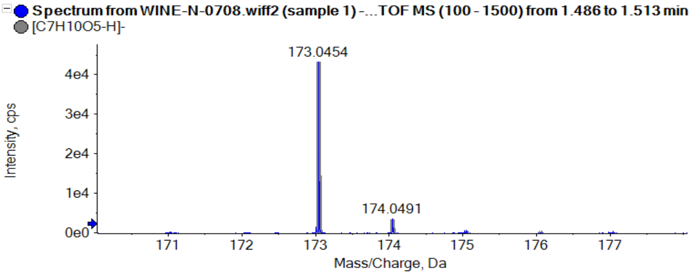

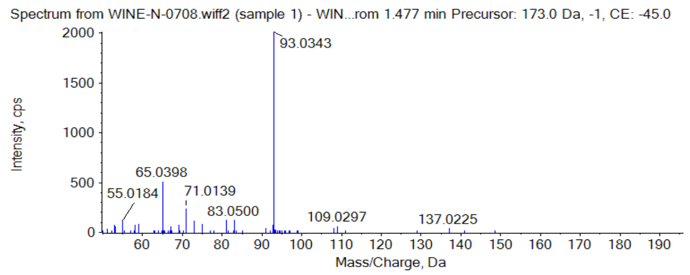

Figure S12. The MS and MS/MS fragments of shikimic acid.

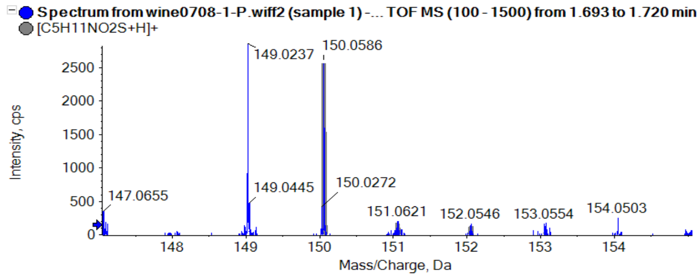

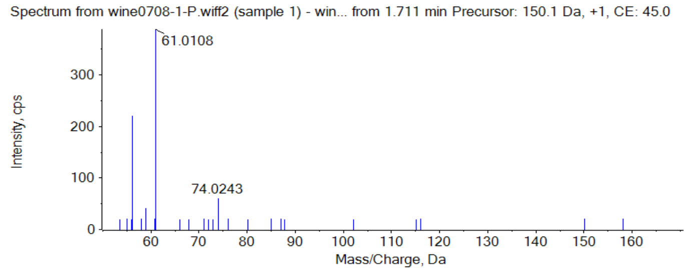

Figure S13. The MS and MS/MS fragments of L-methionine.

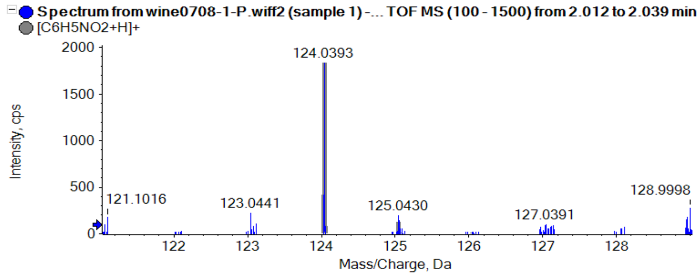

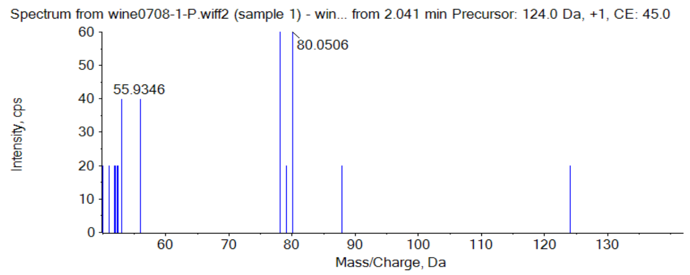

Figure S14. The MS and MS/MS fragments of niacin.

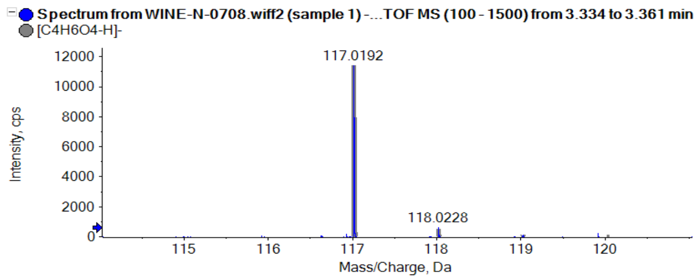

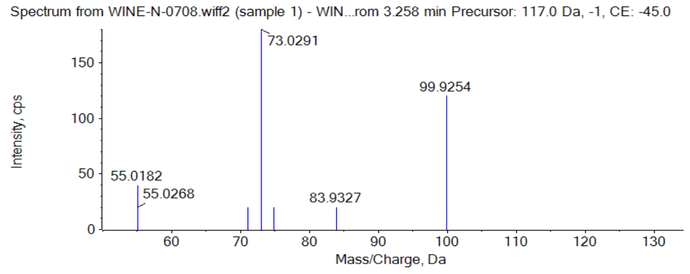

Figure S15. The MS and MS/MS fragments of succinic acid.

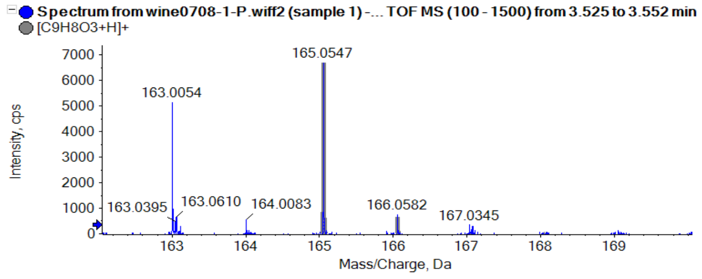

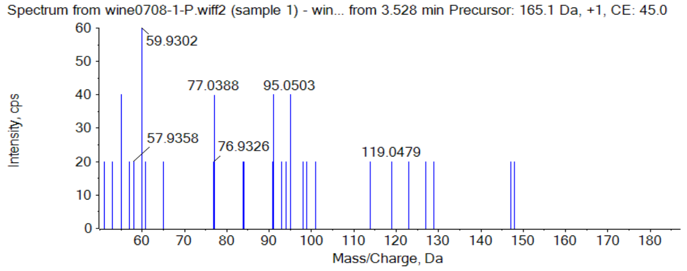

Figure S16. The MS and MS/MS fragments of trans-4-hydroxycinnamic acid.

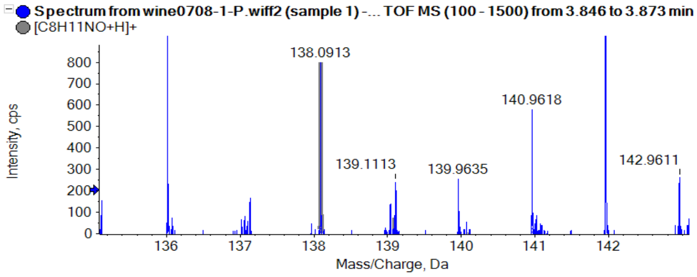

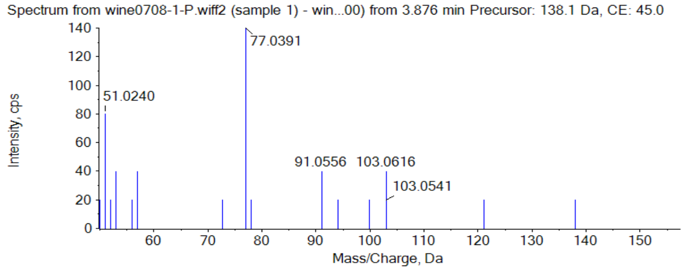

Figure S17. The MS and MS/MS fragments of tyramine.

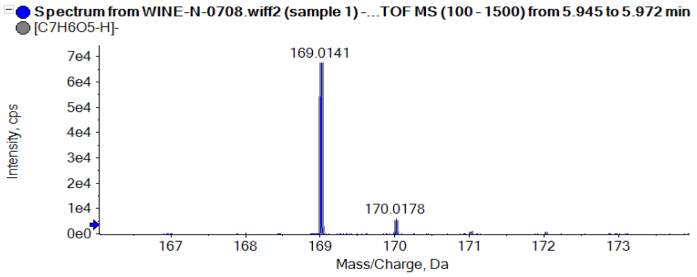

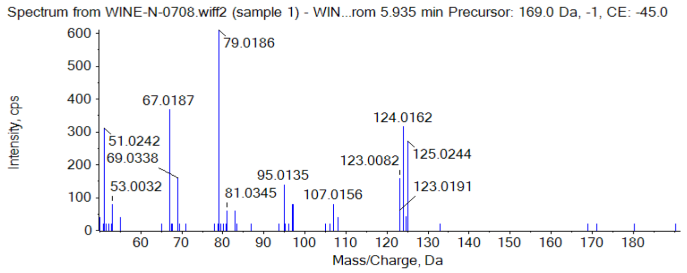

Figure S18. The MS and MS/MS fragments of gallic acid.

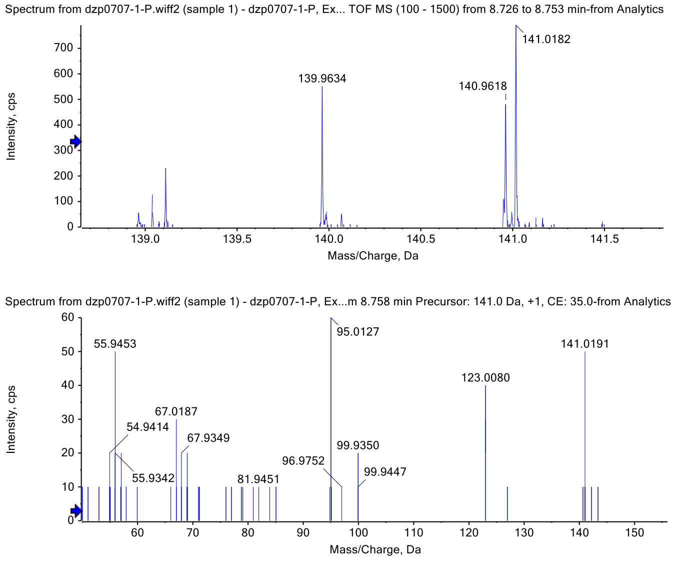

Figure S19. The MS and MS/MS fragments of coumalic acid.

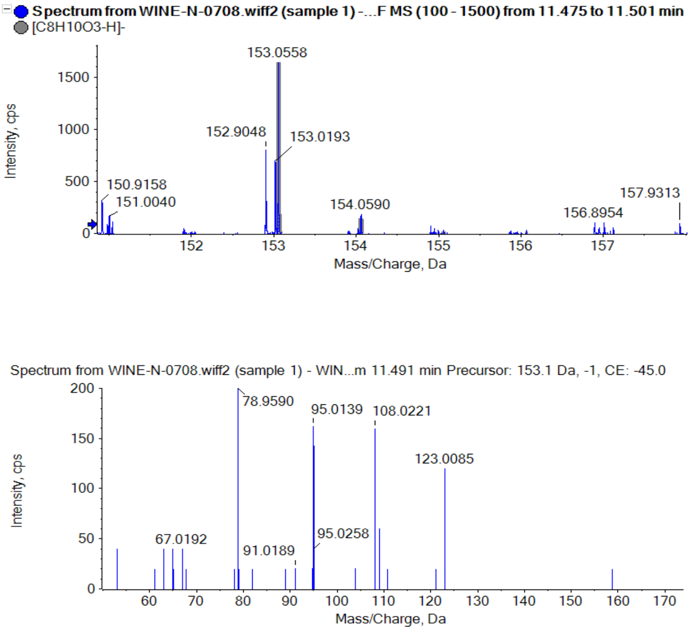

Figure S20. The MS and MS/MS fragments of hydroxytyrosol.

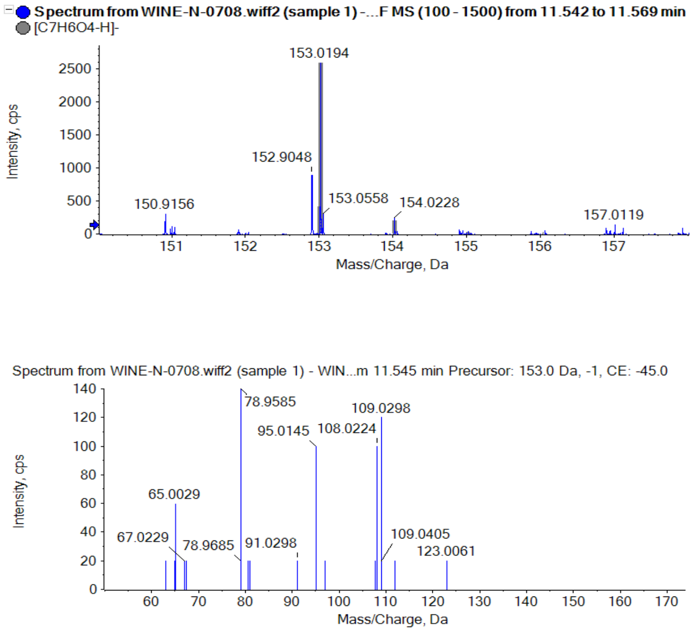

Figure S21. The MS and MS/MS fragments of protocatechuic acid.

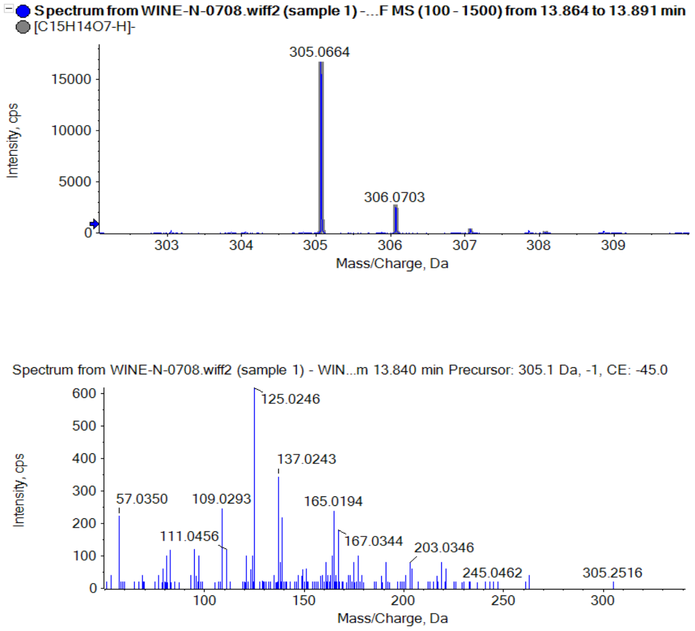

Figure S22. The MS and MS/MS fragments of (+)-gallocatechin.

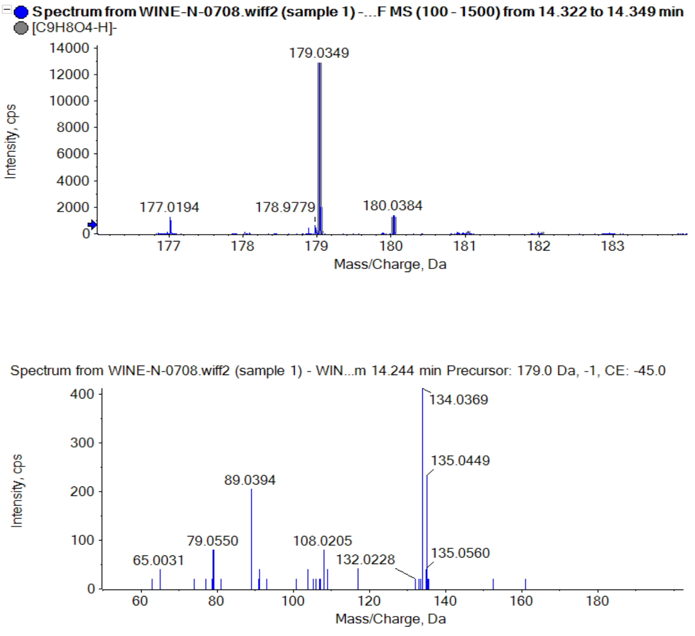

Figure S23. The MS and MS/MS fragments of acetylsalicylic acid.

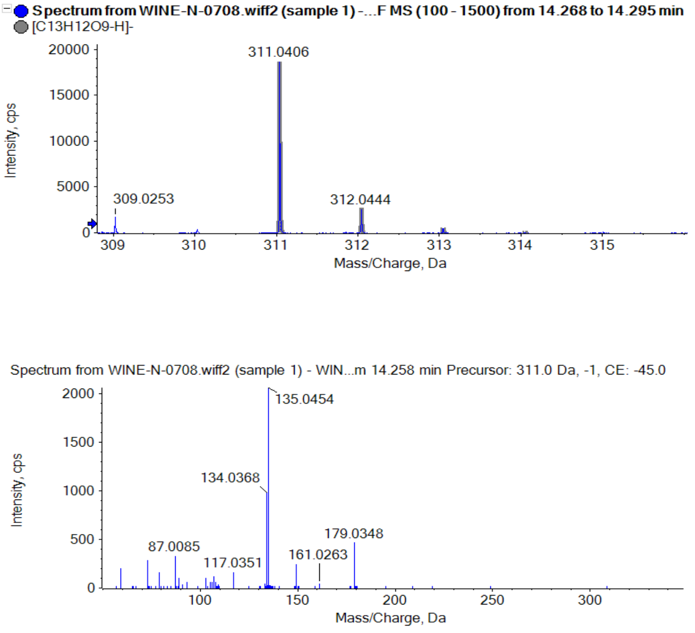

Figure S24. The MS and MS/MS fragments of caftaric acid.

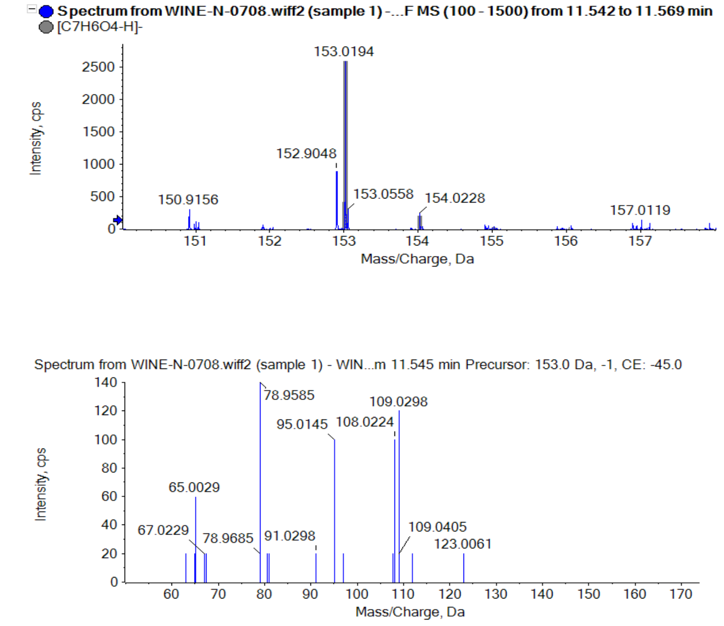

Figure S25. The MS and MS/MS fragments of gentisic acid.

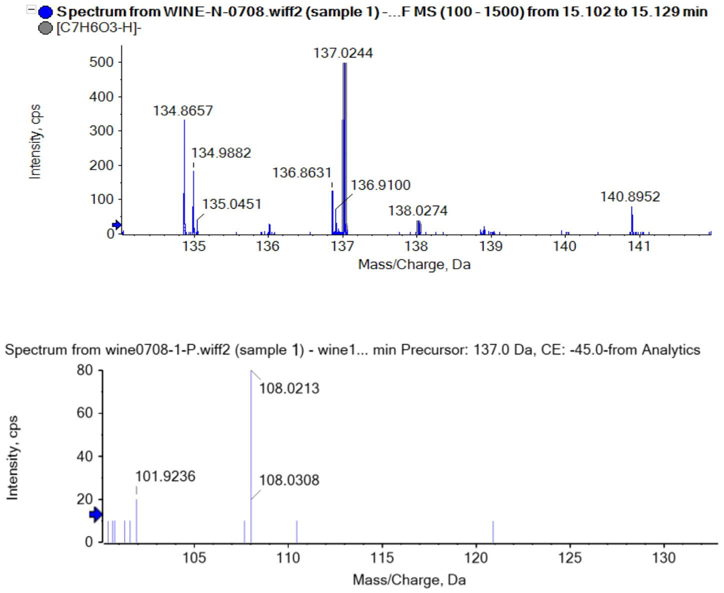

Figure S26. The MS and MS/MS fragments of protocatechualdehyde.

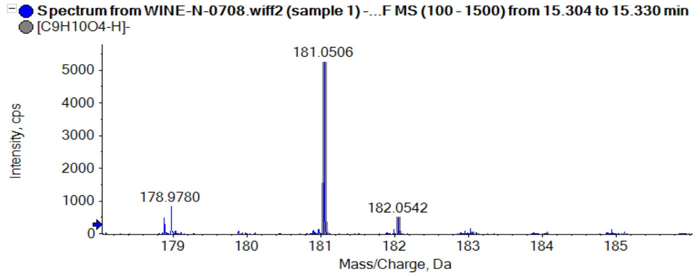

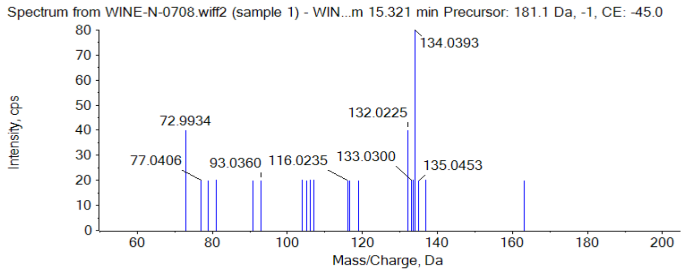

Figure S27. The MS and MS/MS fragments of homovanillic acid.

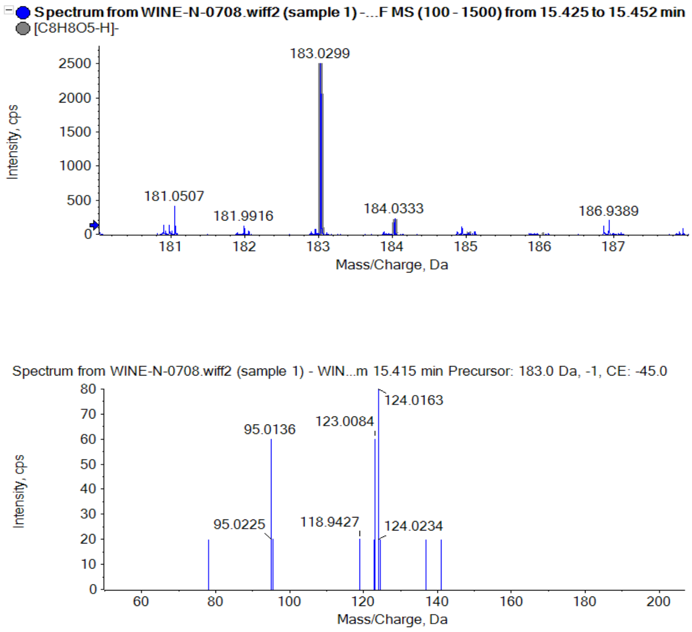

Figure S28. The MS and MS/MS fragments of methyl gallate.

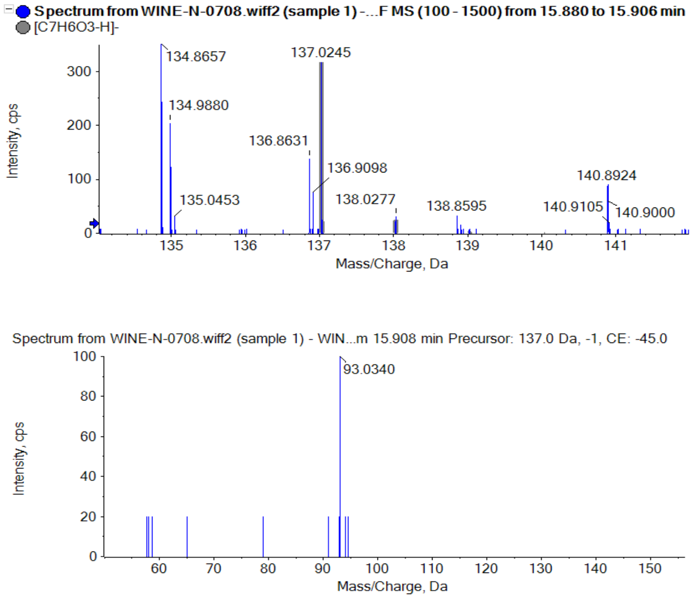

Figure S29. The MS and MS/MS fragments of 4-hydroxybenzoic acid.

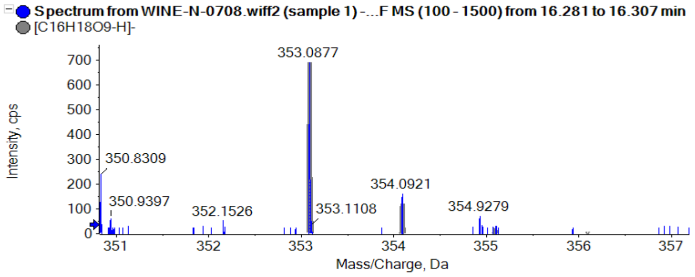

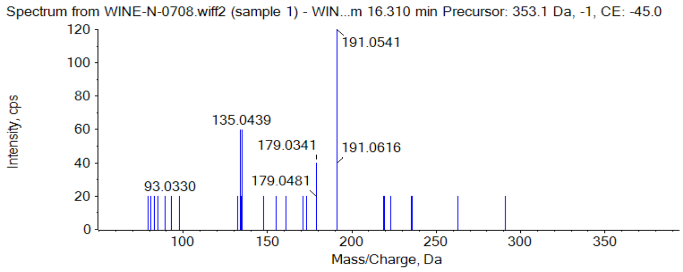

Figure S30. The MS and MS/MS fragments of chlorogenic acid.

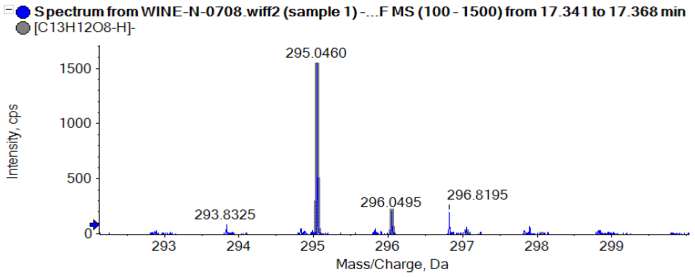

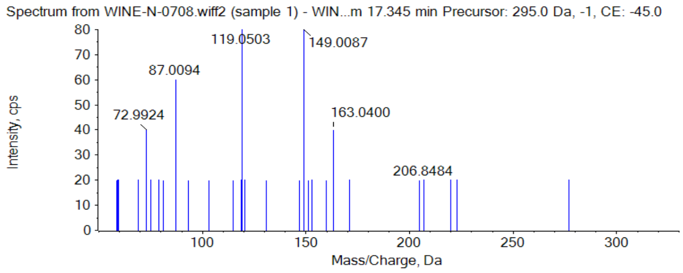

Figure S31. The MS and MS/MS fragments of p-coutaric acid.

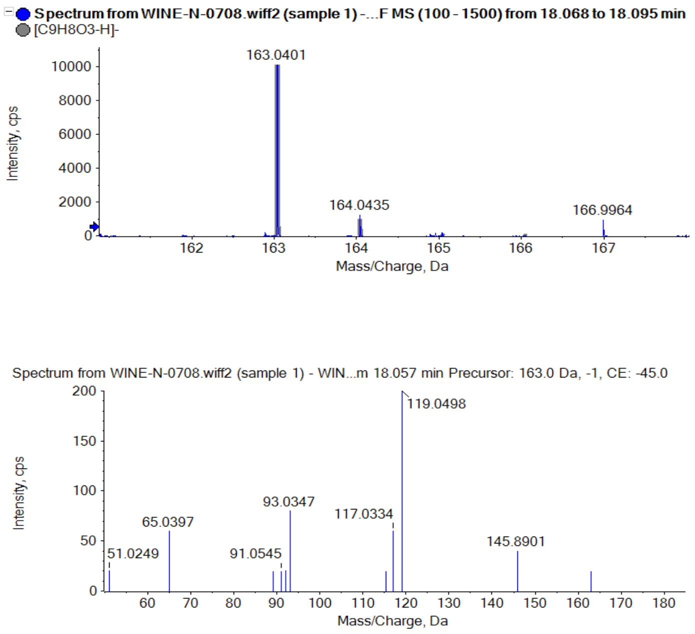

Figure S32. The MS and MS/MS fragments of m-coumaric acid.

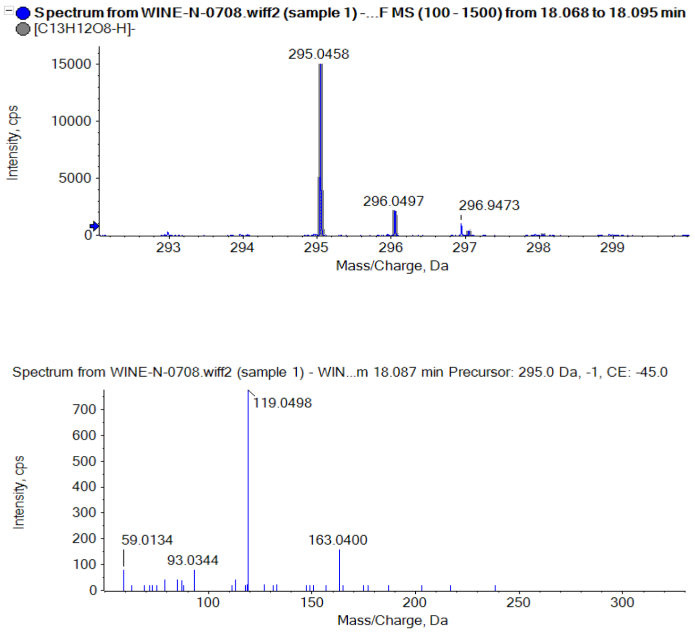

Figure S33. The MS and MS/MS fragments of coutaric acid.

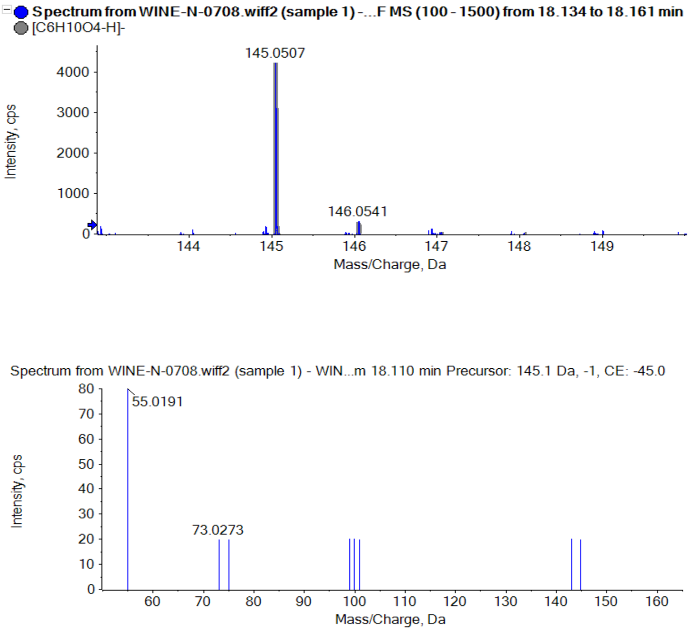

Figure S34. The MS and MS/MS fragments of dimethyl succinate.

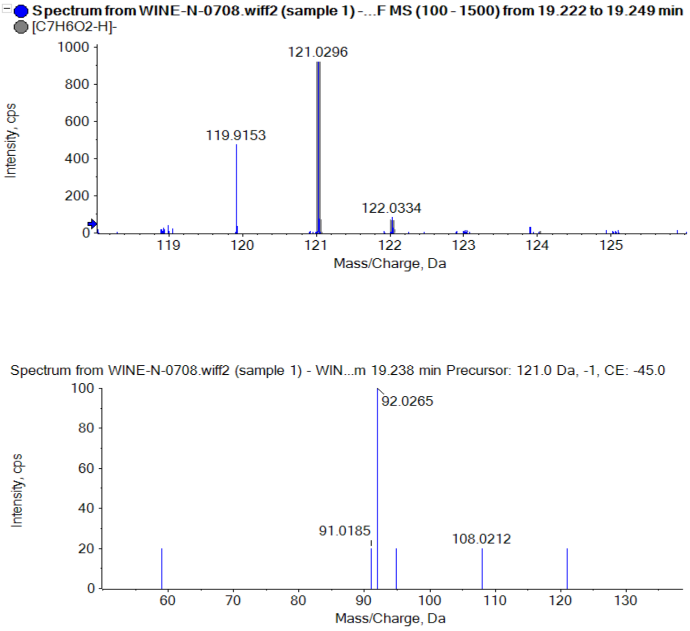

Figure S35. The MS and MS/MS fragments of benzoic acid.

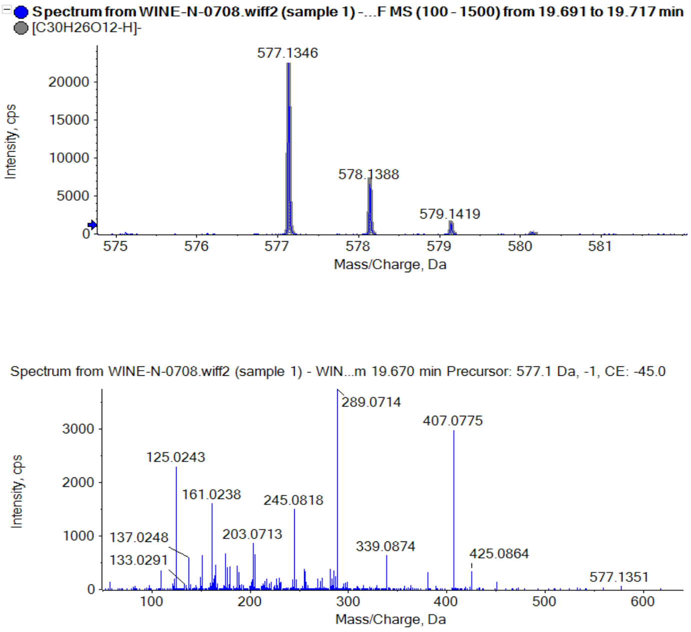

Figure S36. The MS and MS/MS fragments of procyanidin B1.

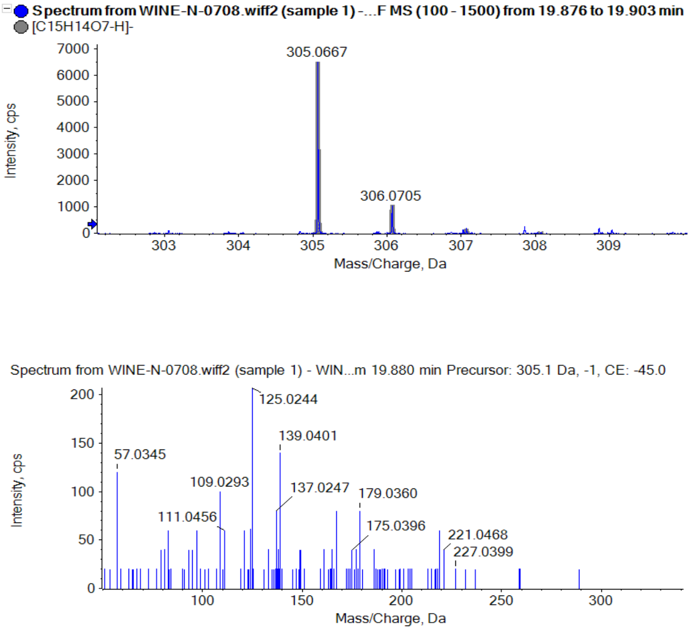

Figure S37. The MS and MS/MS fragments of (-)-epigallocatechin.

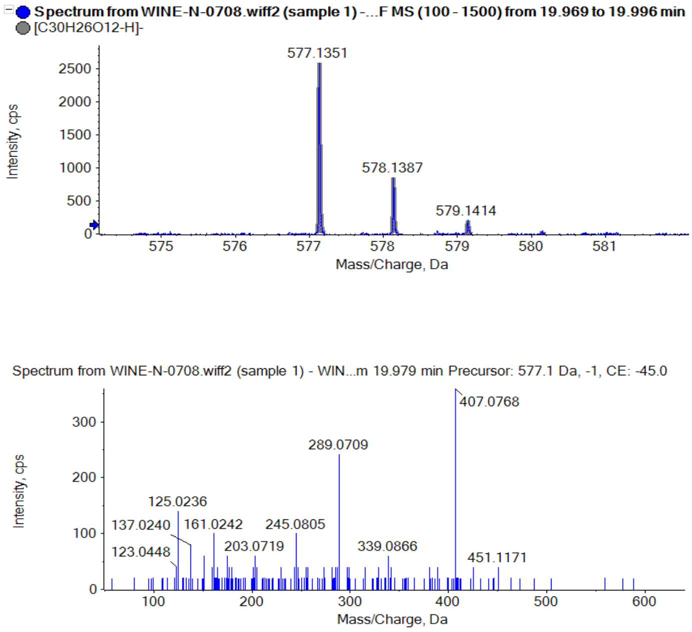

Figure S38. The MS and MS/MS fragments of procyanidin B3.

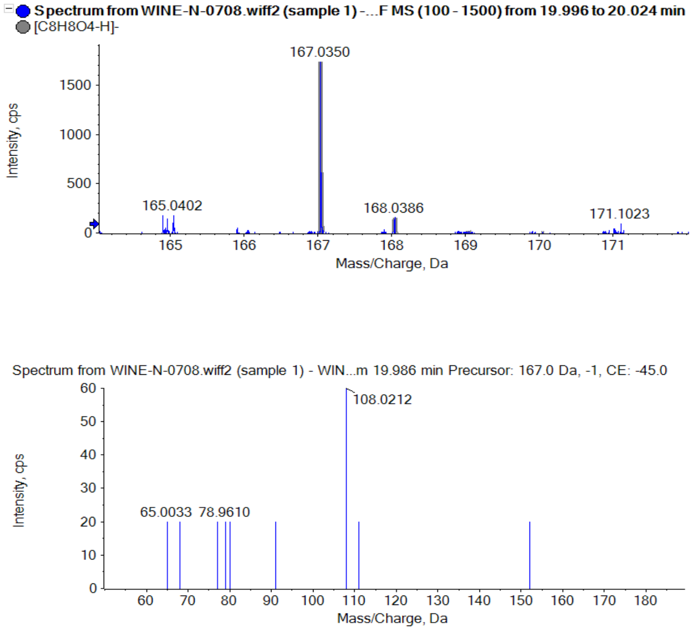

Figure S39. The MS and MS/MS fragments of vanillic acid.

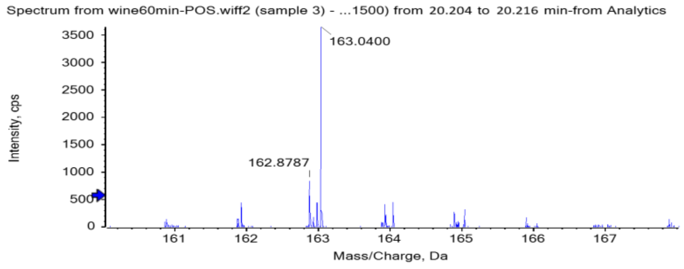

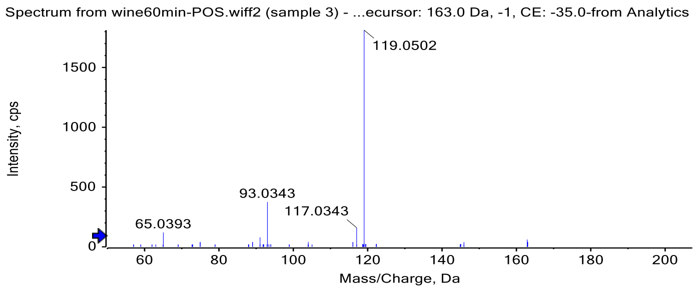

Figure S40. The MS and MS/MS fragments of 2-Hydroxycinnamic acid.

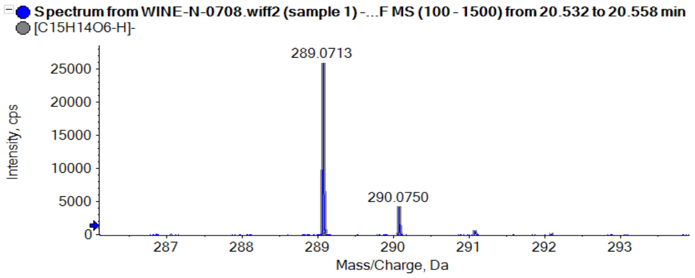

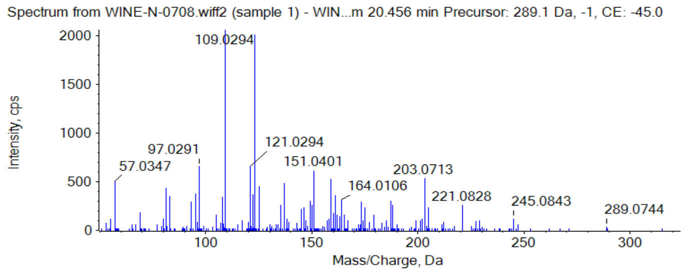

Figure S41. The MS and MS/MS fragments of catechin.

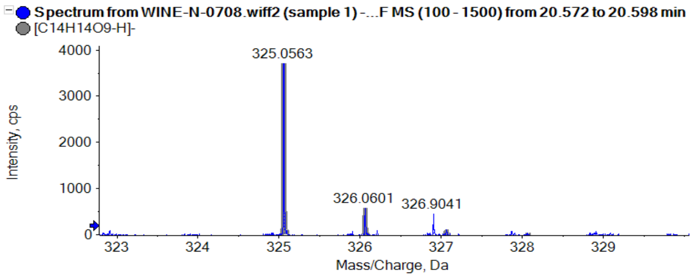

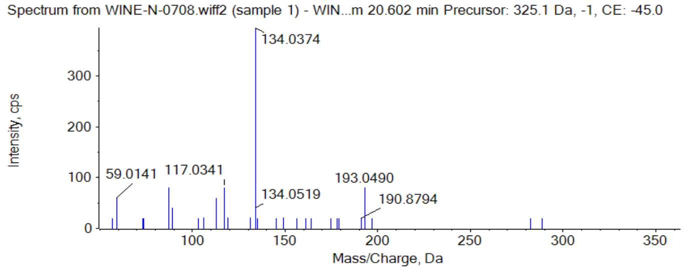

Figure S42. The MS and MS/MS fragments of fertaric acid.

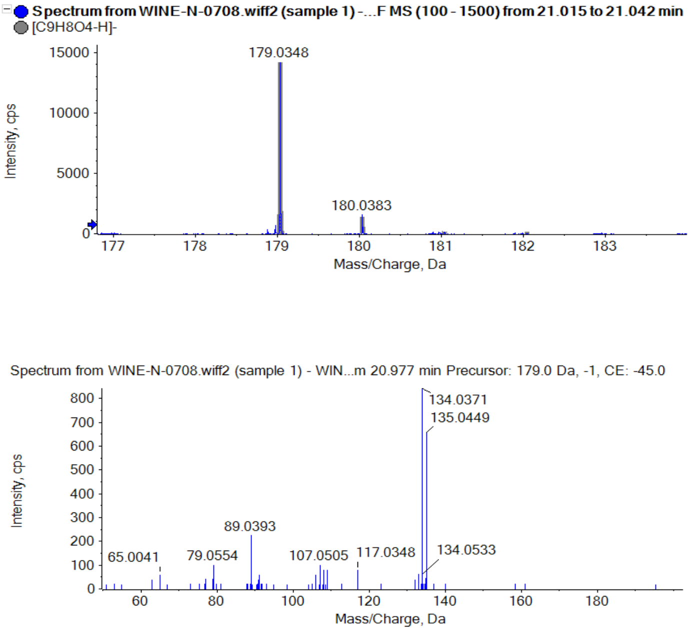

Figure S43. The MS and MS/MS fragments of caffeic acid.

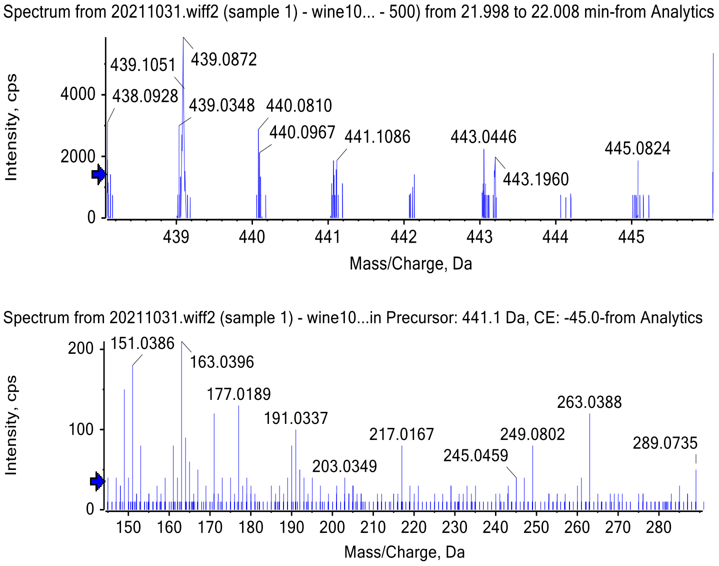

Figure S44. The MS and MS/MS fragments of epicatechin gallate.

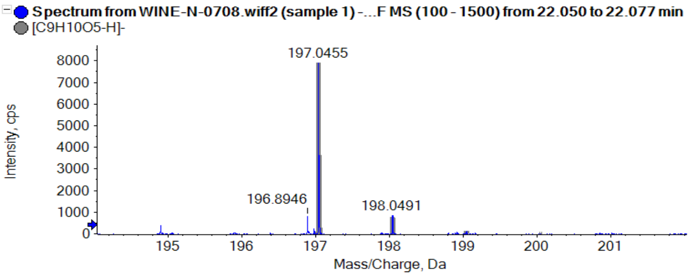

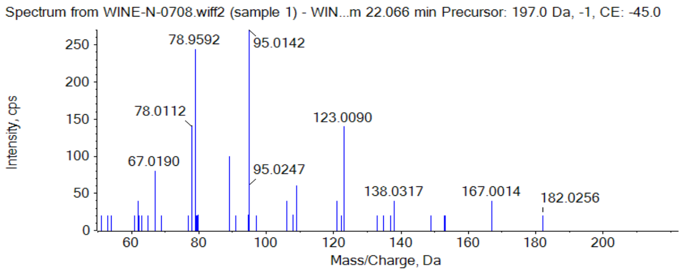

Figure S45. The MS and MS/MS fragments of syringic acid.

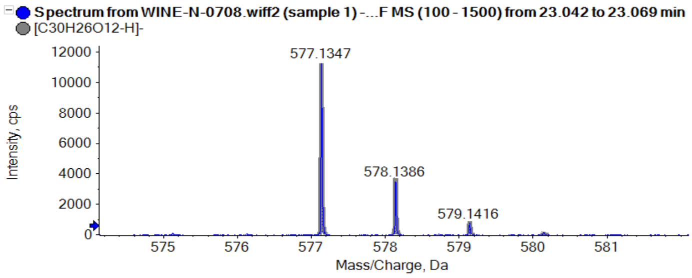

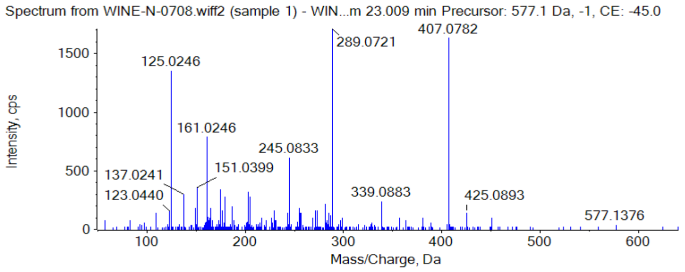

Figure S46. The MS and MS/MS fragments of procyanidin B2.

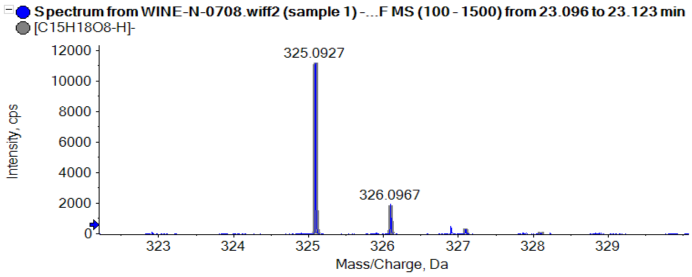

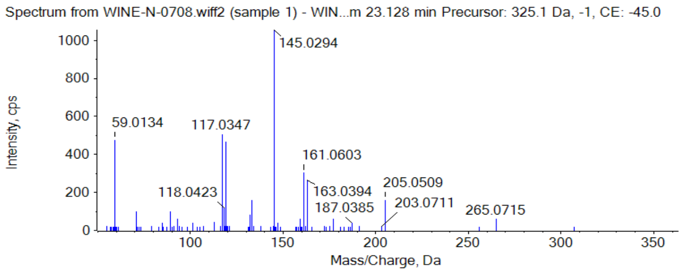

Figure S47. The MS and MS/MS fragments of p-coumaric acid glucoside.

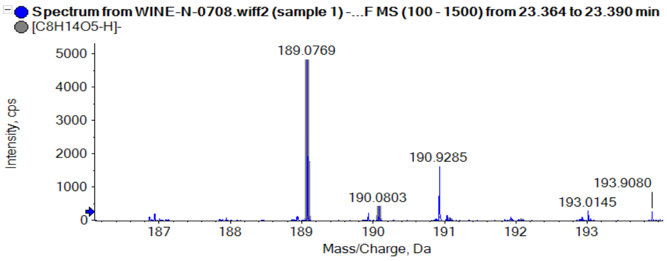

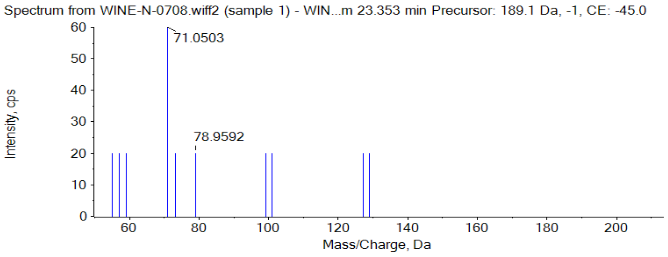

Figure S48. The MS and MS/MS fragments of diethyl malate.

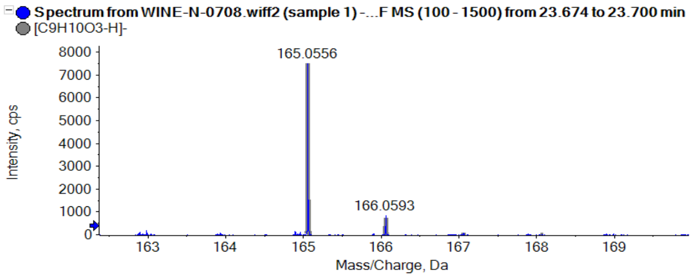

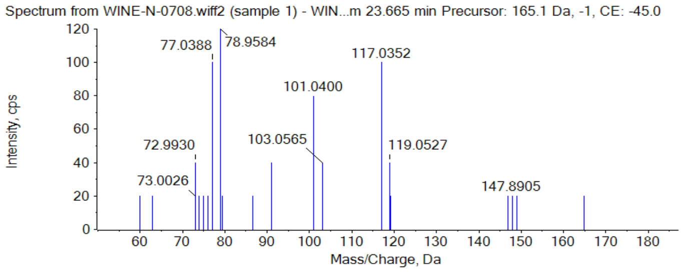

Figure S49. The MS and MS/MS fragments of desaminotyrosine.

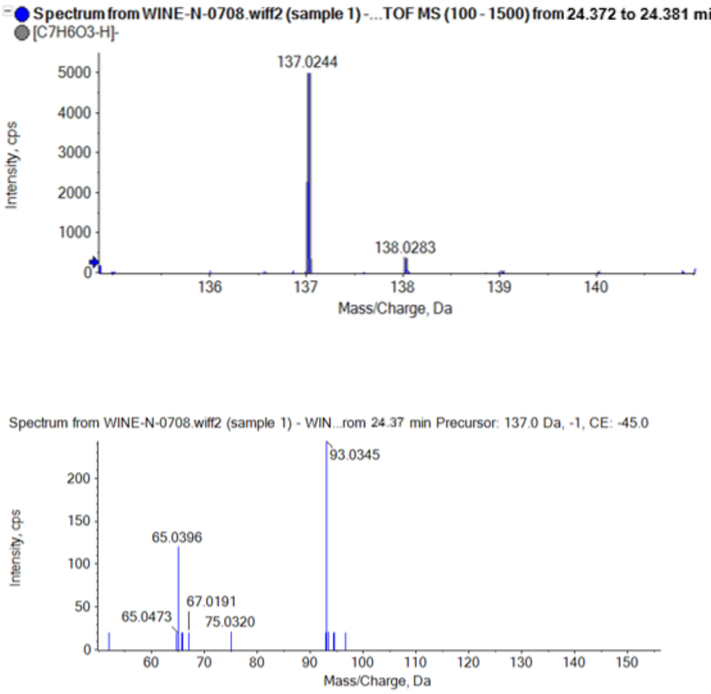

Figure S50. The MS and MS/MS fragments of 2-hydroxybenzoic acid.

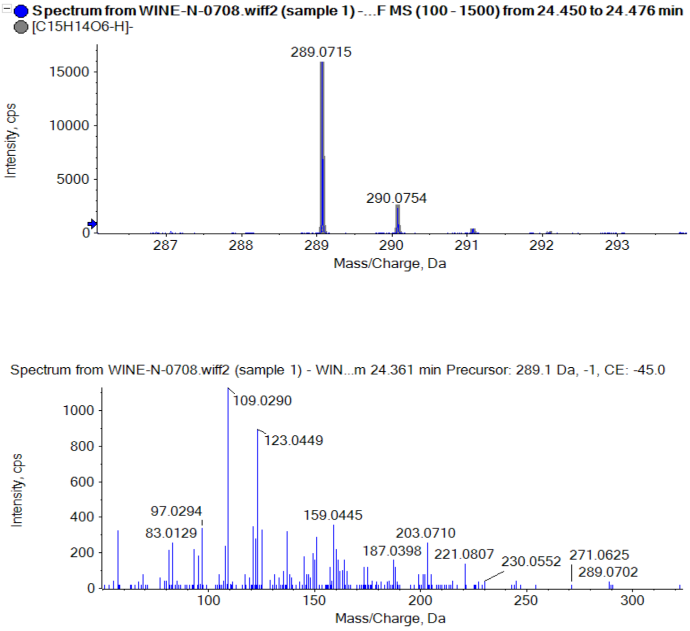

Figure S51. The MS and MS/MS fragments of epicatechin.

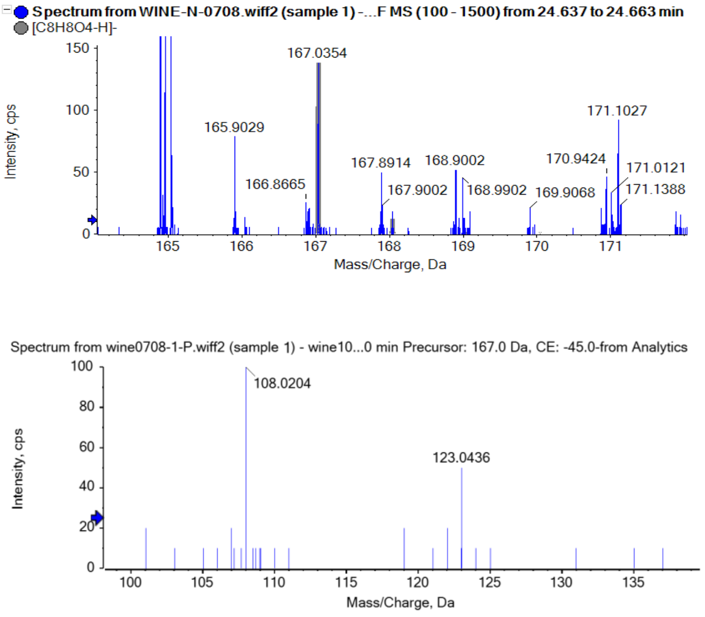

Figure S52. The MS and MS/MS fragments of 3-hydroxy-4-methoxybenzoic acid.

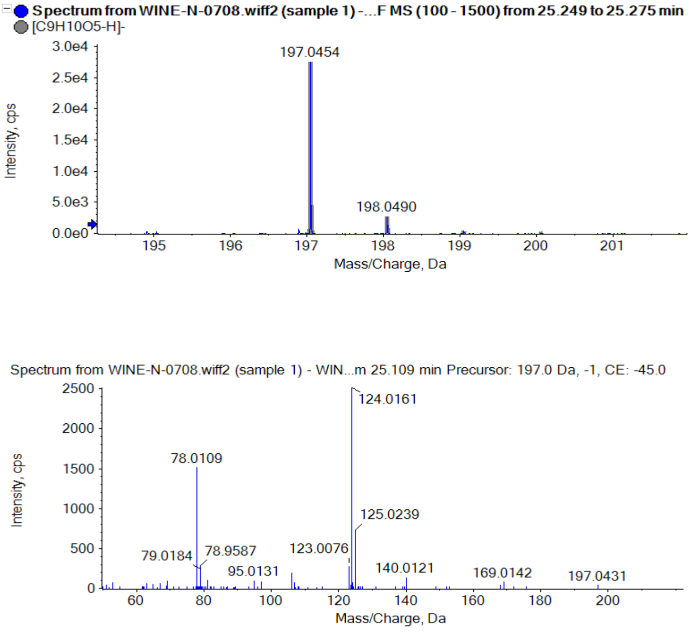

Figure S53. The MS and MS/MS fragments of ethyl gallate.

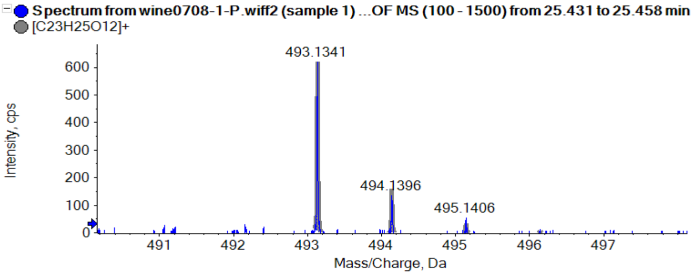

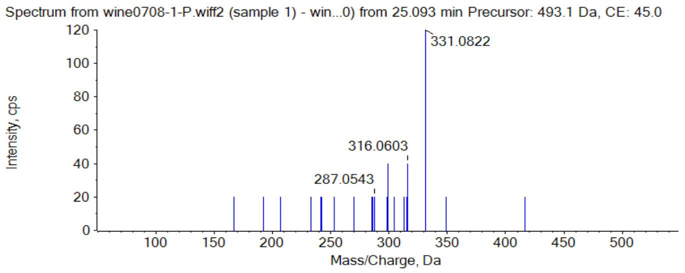

Figure S54. The MS and MS/MS fragments of malvidin-3-O-glucoside.

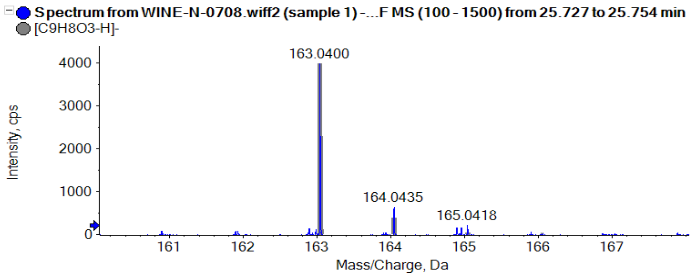

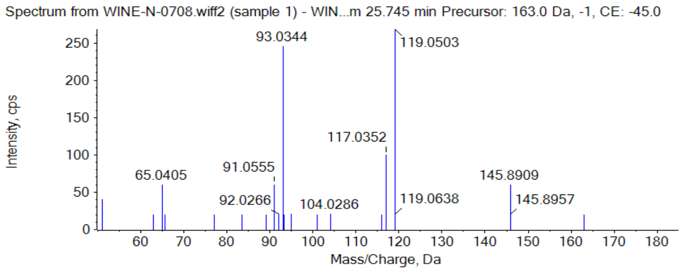

Figure S55. The MS and MS/MS fragments of p-coumaric acid.

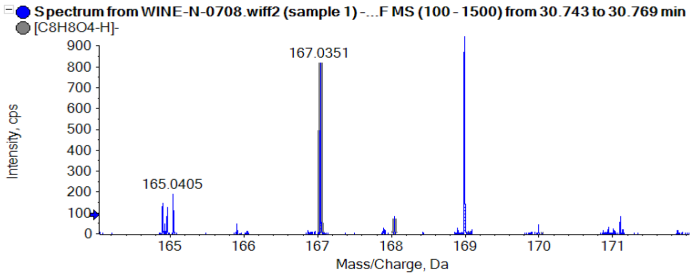

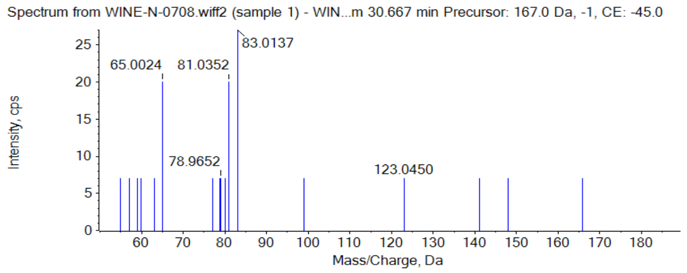

Figure S56. The MS and MS/MS fragments of homogentisic acid.

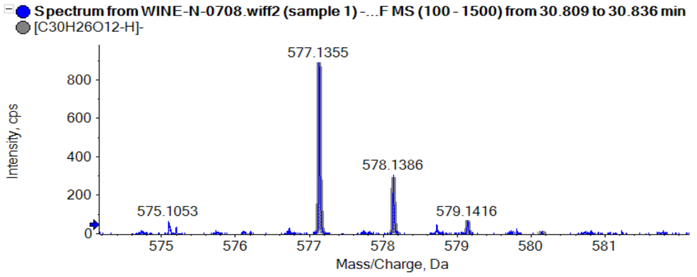

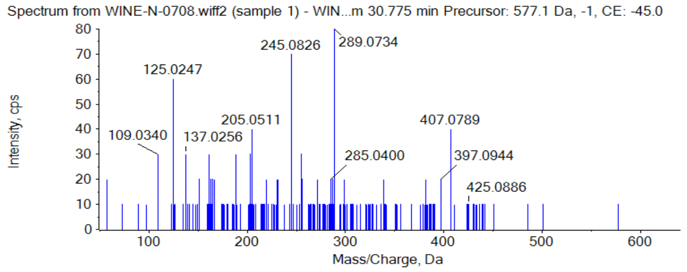

Figure S57. The MS and MS/MS fragments of procyanidin B7.

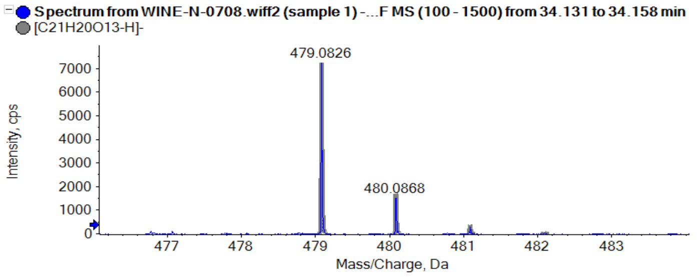

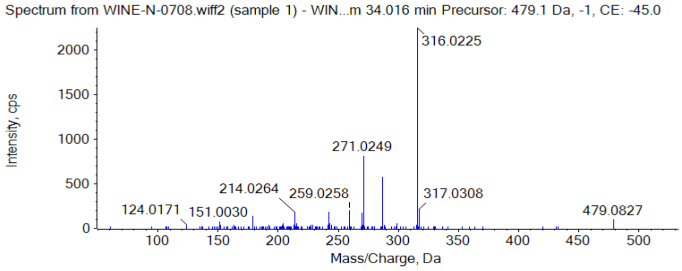

Figure S58. The MS and MS/MS fragments of myricetin-3-O-galactoside.

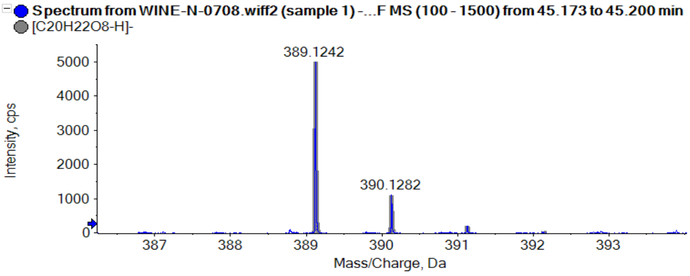

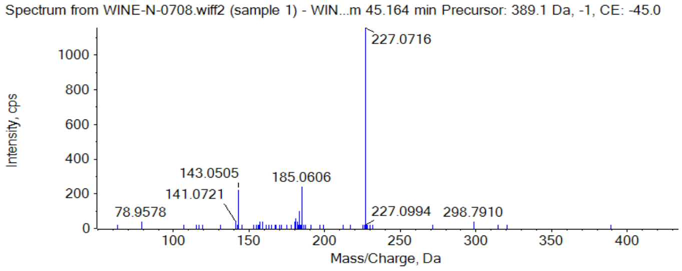

Figure S59. The MS and MS/MS fragments of trans-piceid.

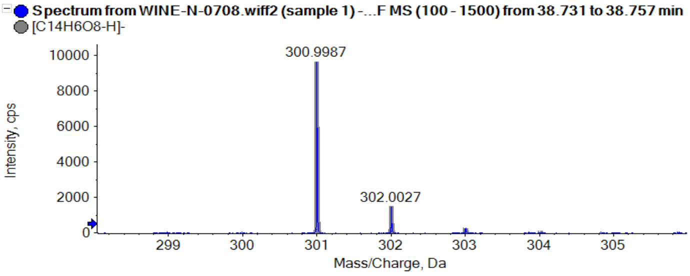

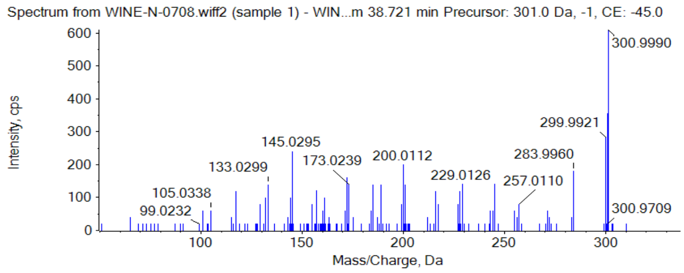

Figure S60. The MS and MS/MS fragments of ellagic acid.

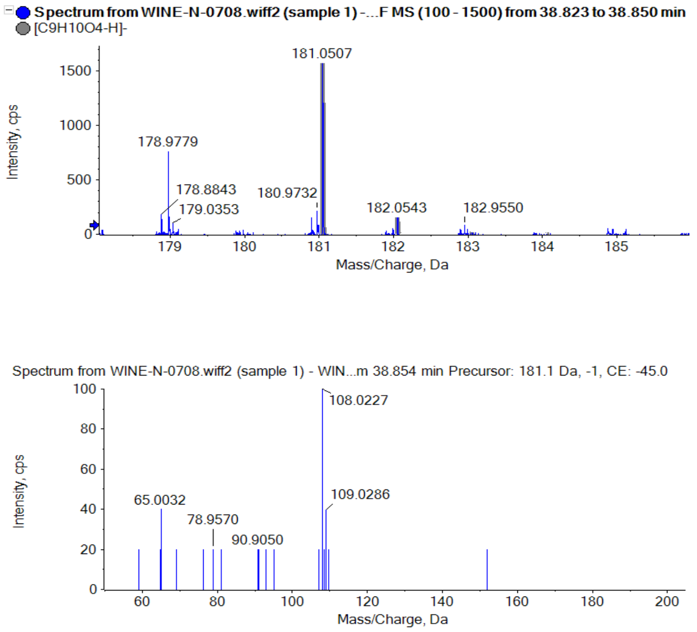

Figure S61. The MS and MS/MS fragments of dihydrocaffeic acid.

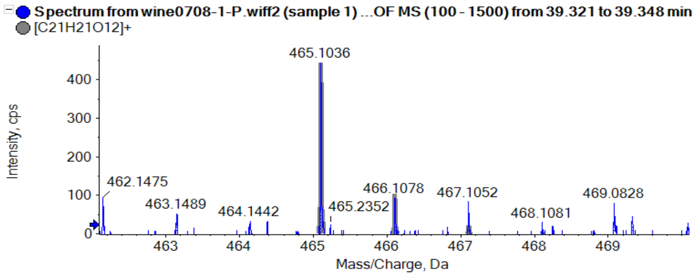

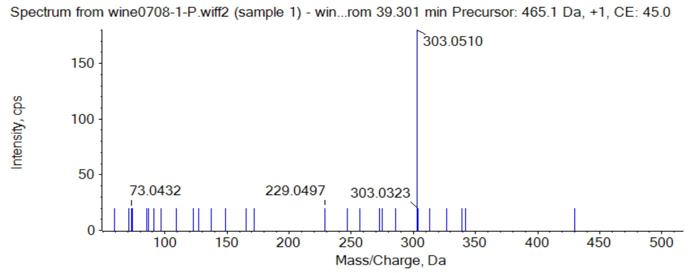

Figure S62. The MS and MS/MS fragments of delphinidin-3-O-glucoside.

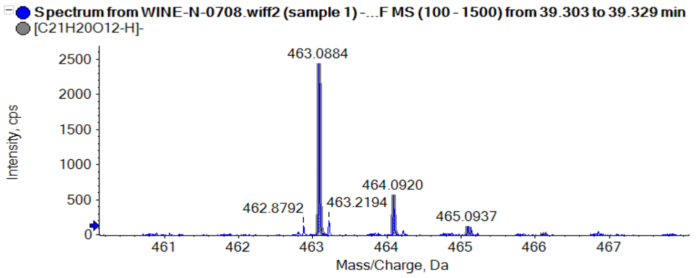

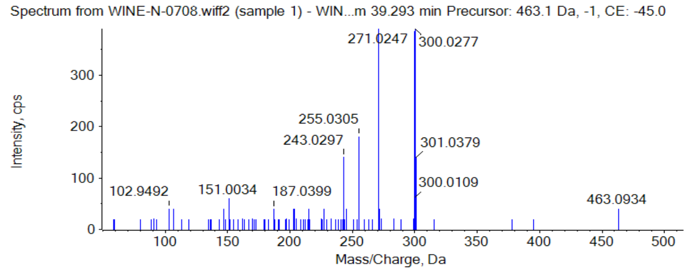

Figure S63. The MS and MS/MS fragments of hyperoside.

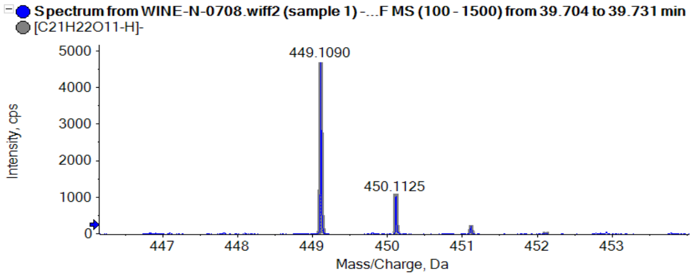

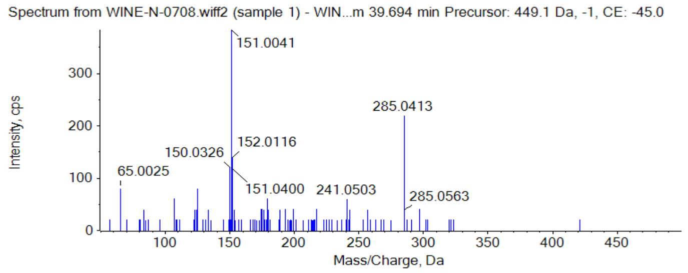

Figure S64. The MS and MS/MS fragments of quercitrin.

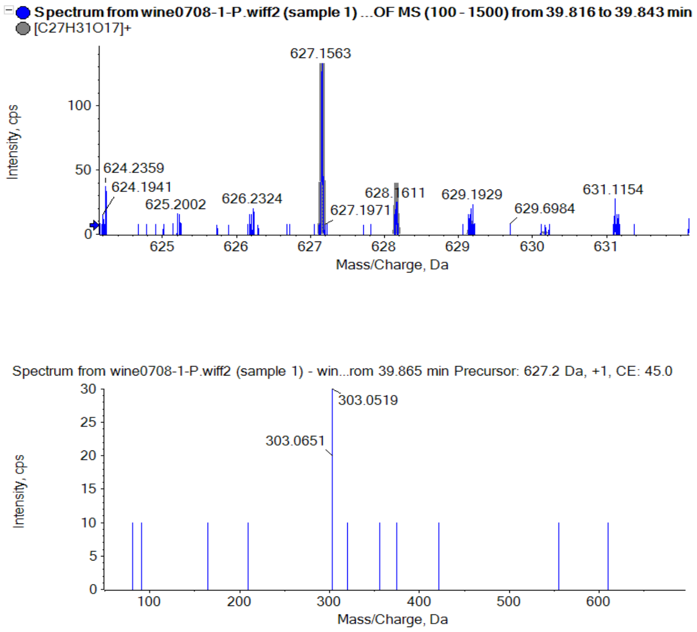

Figure S65. The MS and MS/MS fragments of delphinidin-3,5-O-diglucoside.

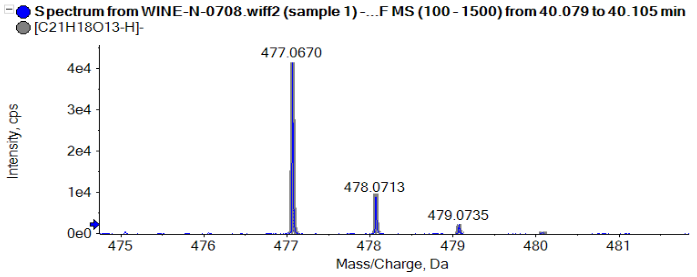

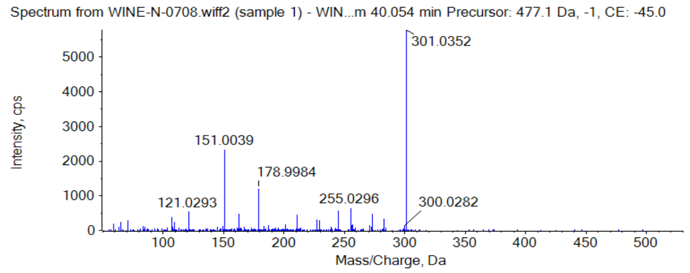

Figure S66. The MS and MS/MS fragments of quercetin-3-O-glucuronide.

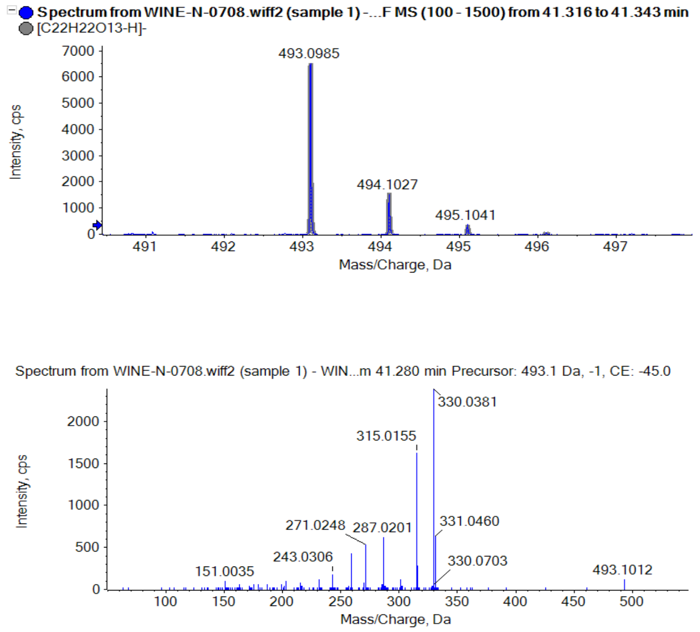

Figure S67. The MS and MS/MS fragments of laricitrin-3-O-glucoside.

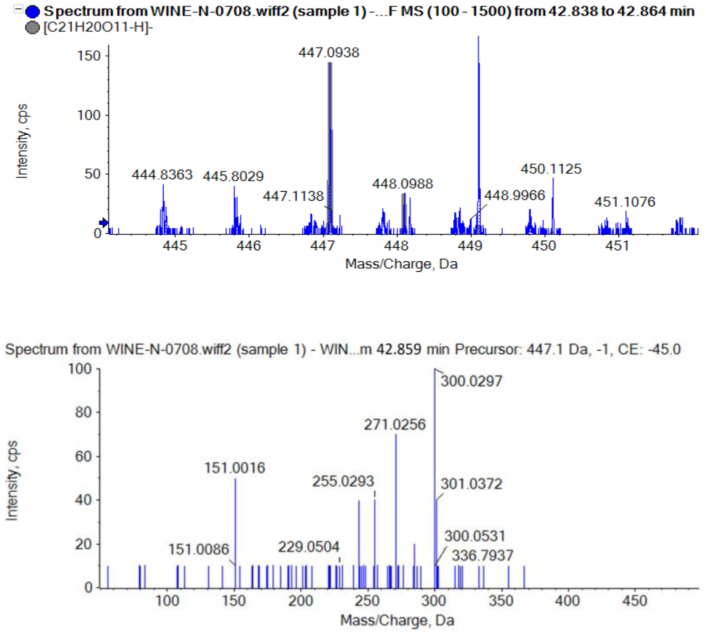

Figure S68. The MS and MS/MS fragments of luteolin -3'-glucoside.

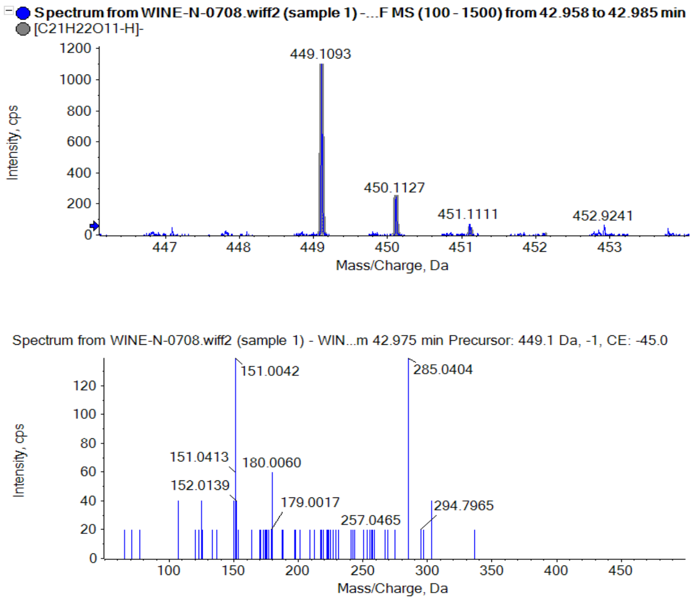

Figure S69. The MS and MS/MS fragments of astilbin.

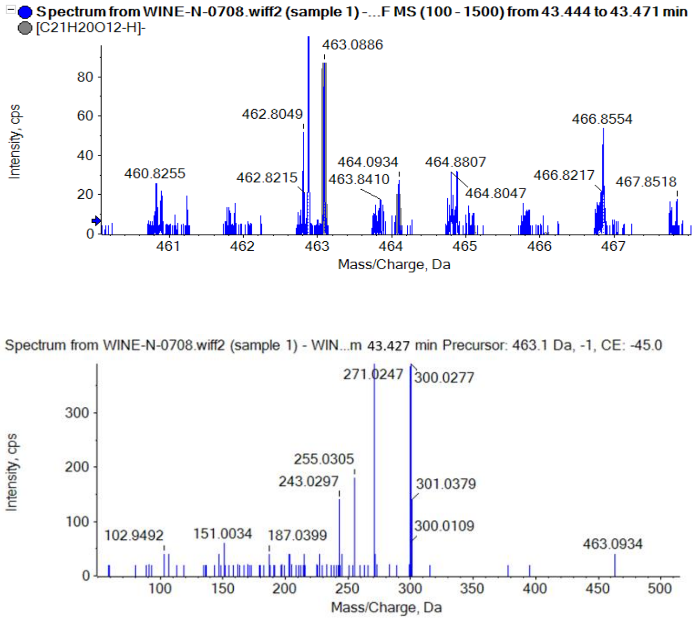

Figure S70. The MS and MS/MS fragments of isoquercitrin.

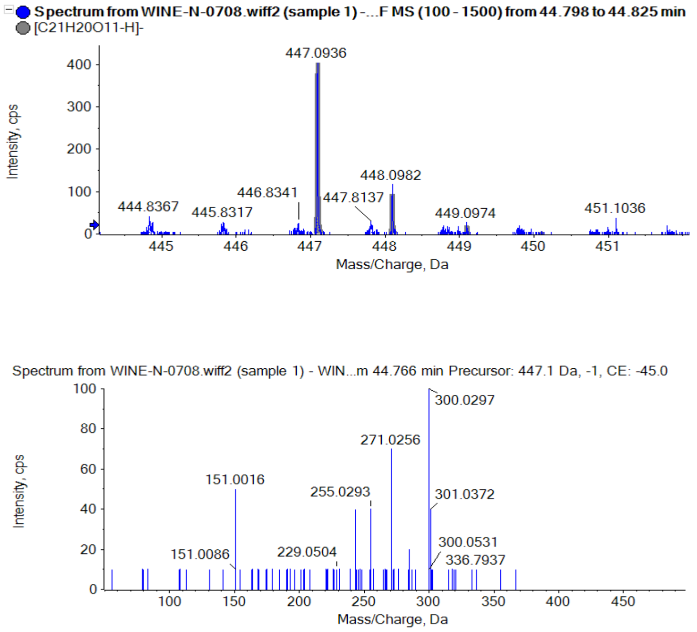

Figure S71. The MS and MS/MS fragments of astragalin.

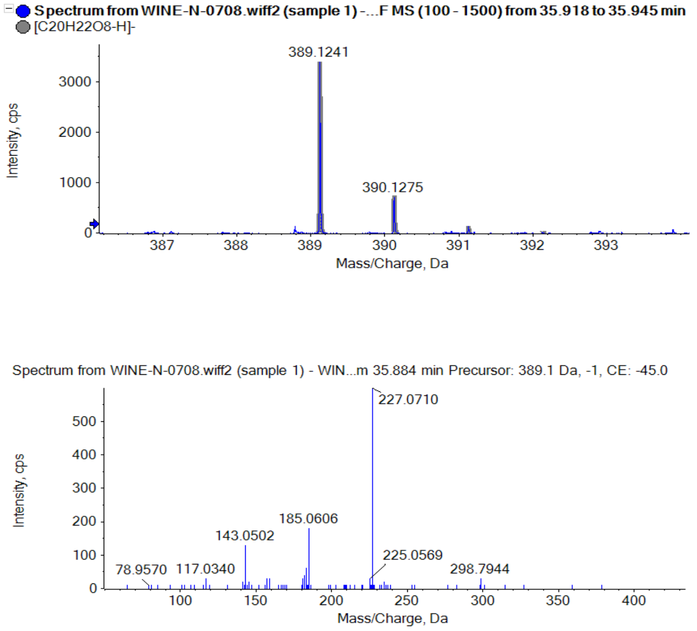

Figure S72. The MS and MS/MS fragments of cis-piceid.

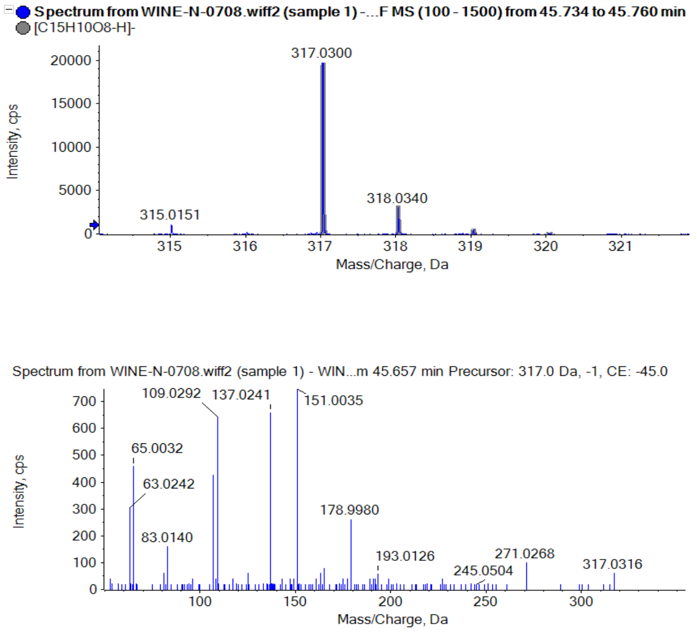

Figure S73. The MS and MS/MS fragments of myricetin.

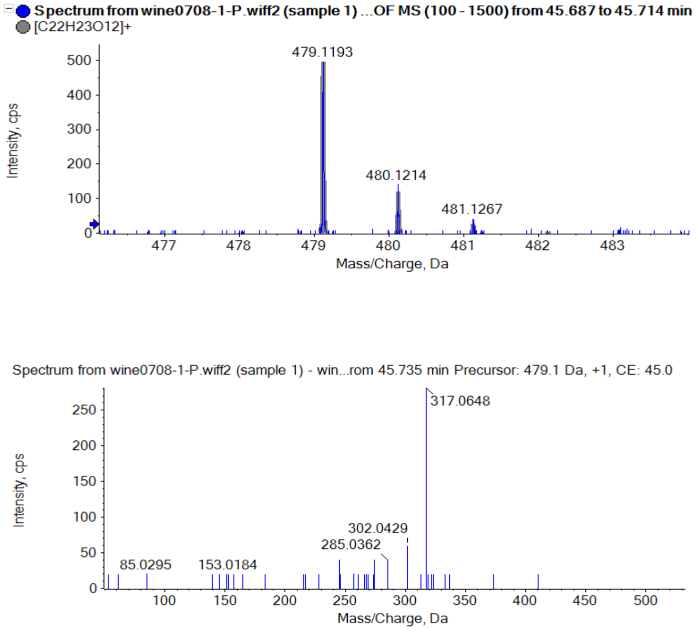

Figure S74. The MS and MS/MS fragments of petunidin-3-O-glucoside.

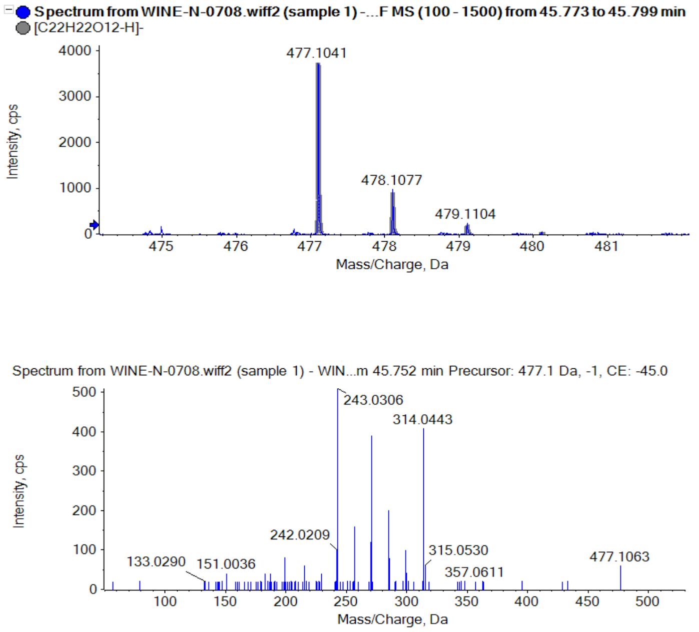

Figure S75. The MS and MS/MS fragments of isorhamnetin-3-O-glucoside.

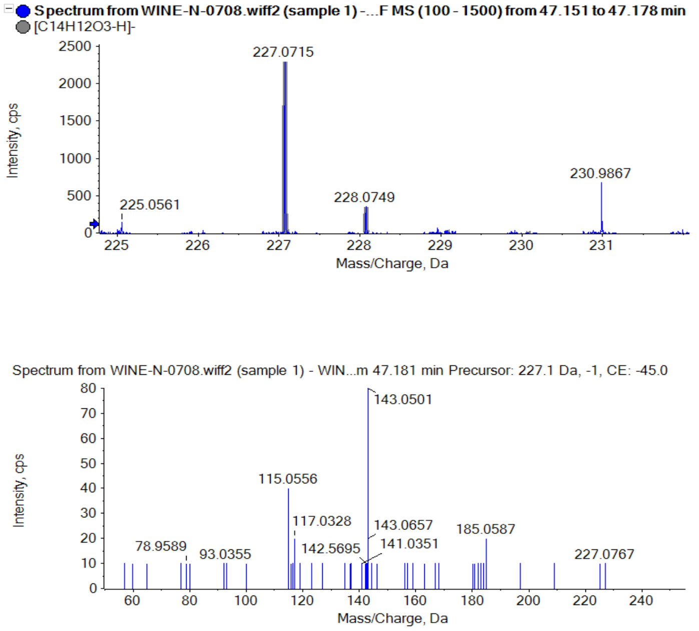

Figure S76. The MS and MS/MS fragments of trans-resveratrol.

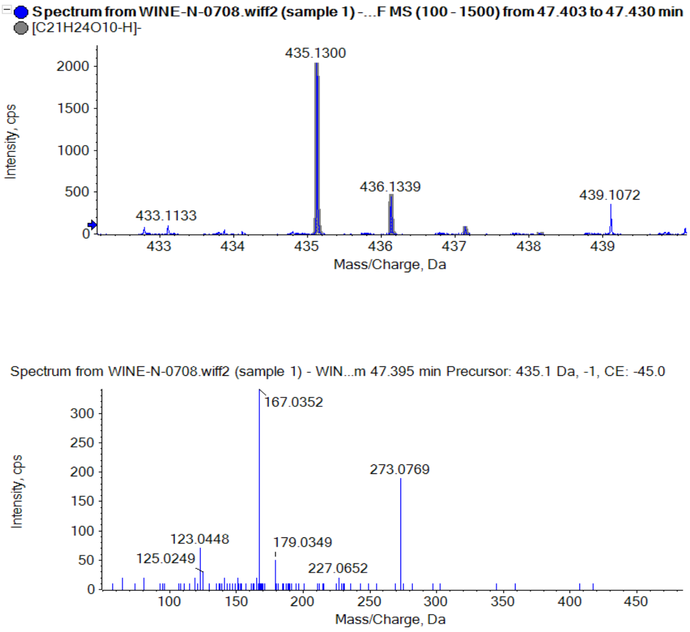

Figure S77. The MS and MS/MS fragments of phloridzin.

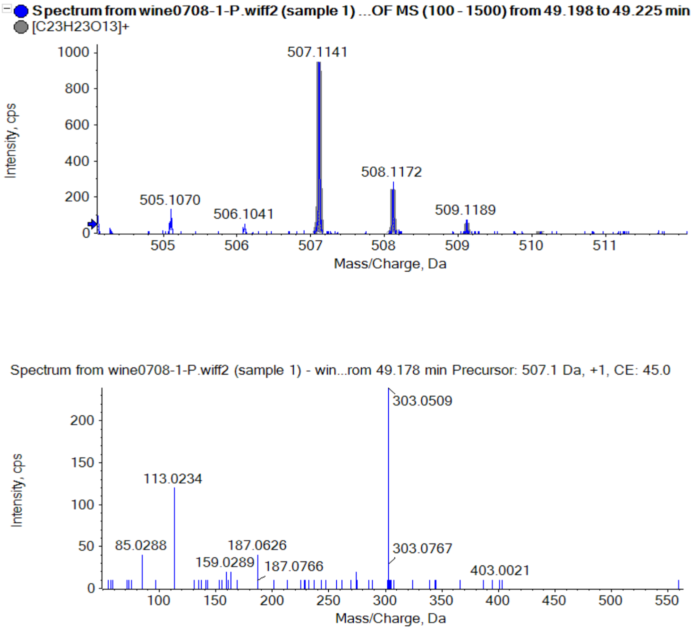

Figure S78. The MS and MS/MS fragments of delphindin-3-O-(6-O-acetylglucoside).

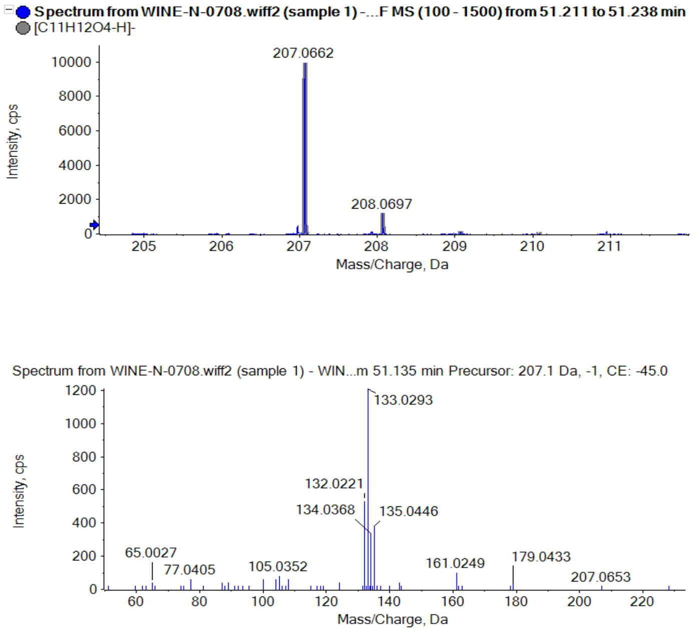

Figure S79. The MS and MS/MS fragments of ethyl caffeate.

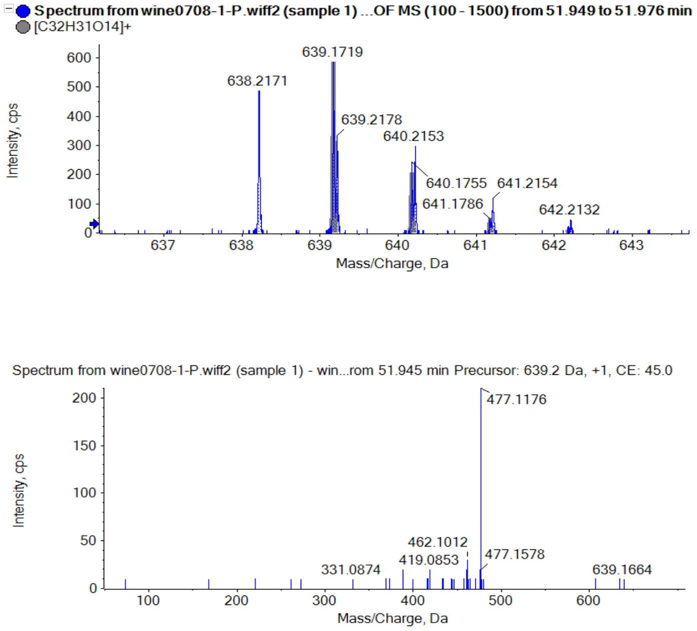

Figure S80. The MS and MS/MS fragments of malvidin-3-O-(6-O-coumaroylglucoside).

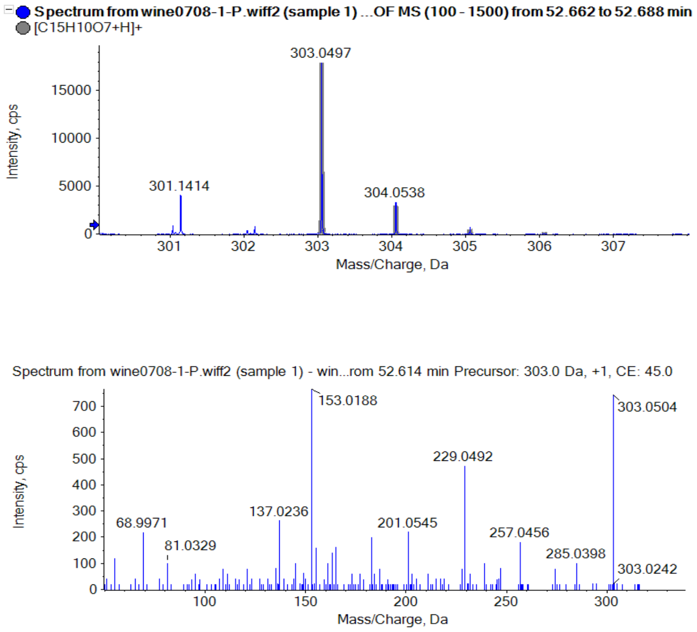

Figure S81. The MS and MS/MS fragments of morin.

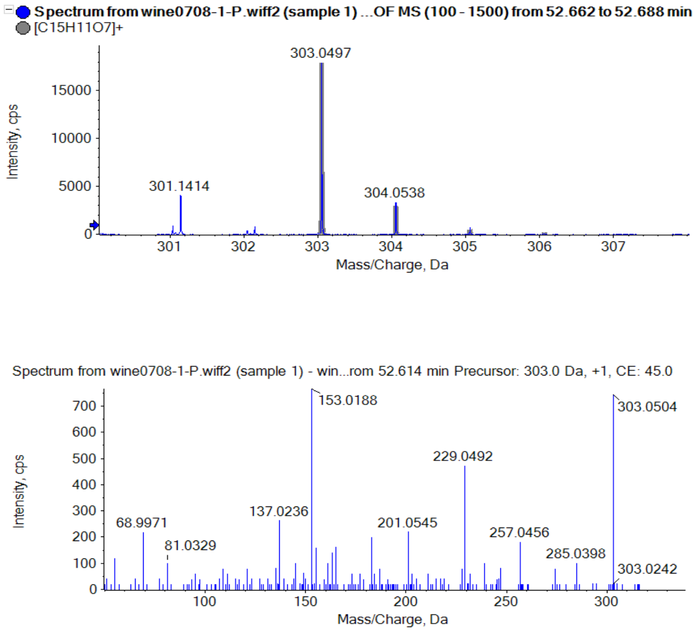

Figure S82. The MS and MS/MS fragments of delphinidin.

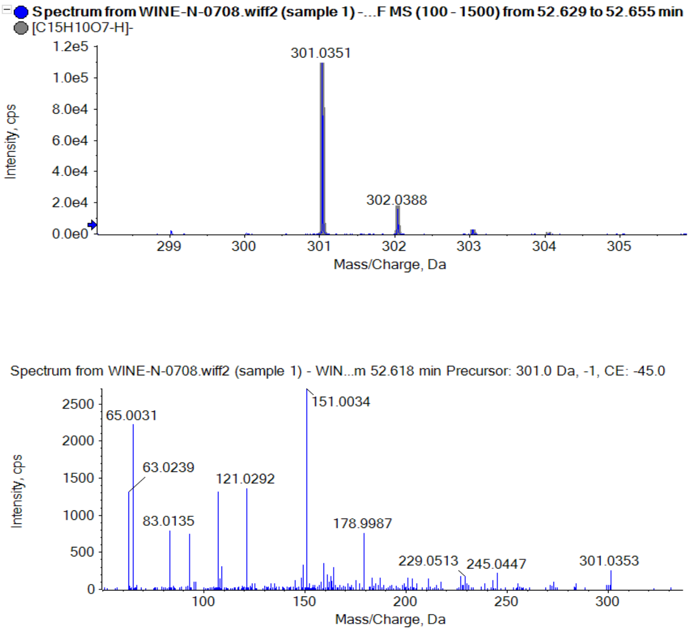

Figure S83. The MS and MS/MS fragments of quercetin.

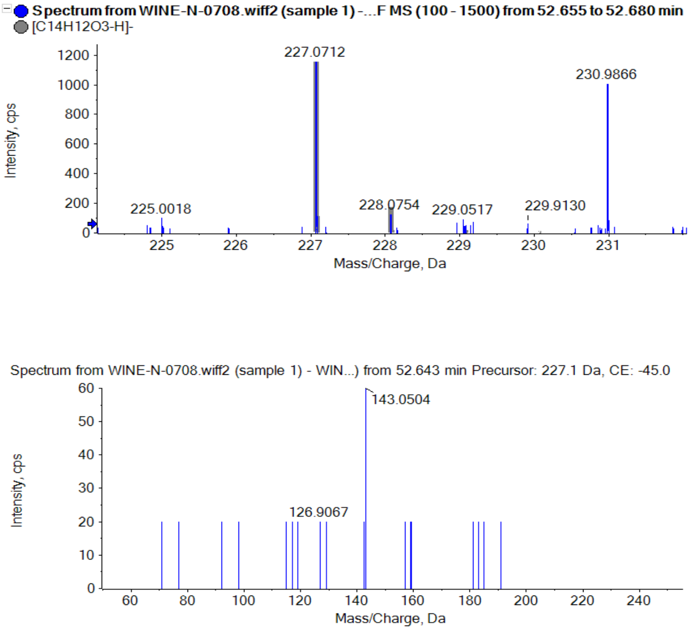

Figure S84. The MS and MS/MS fragments of resveratrol.

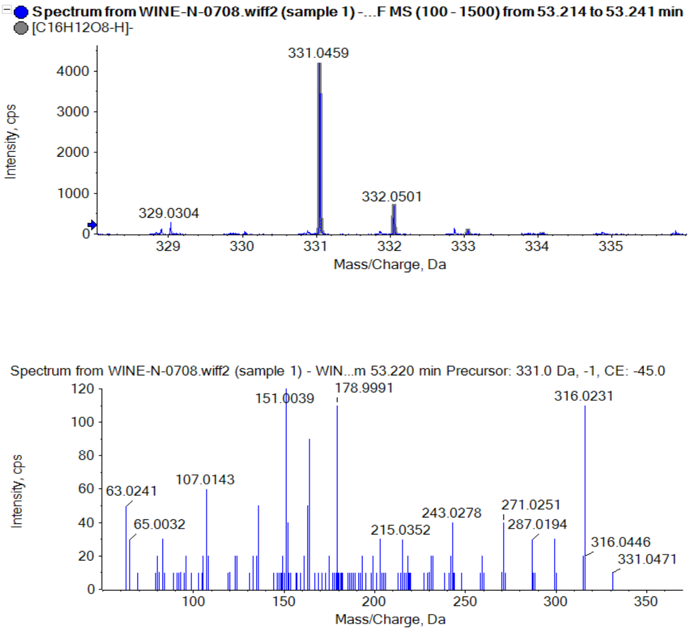

Figure S85. The MS and MS/MS fragments of laricitrin.

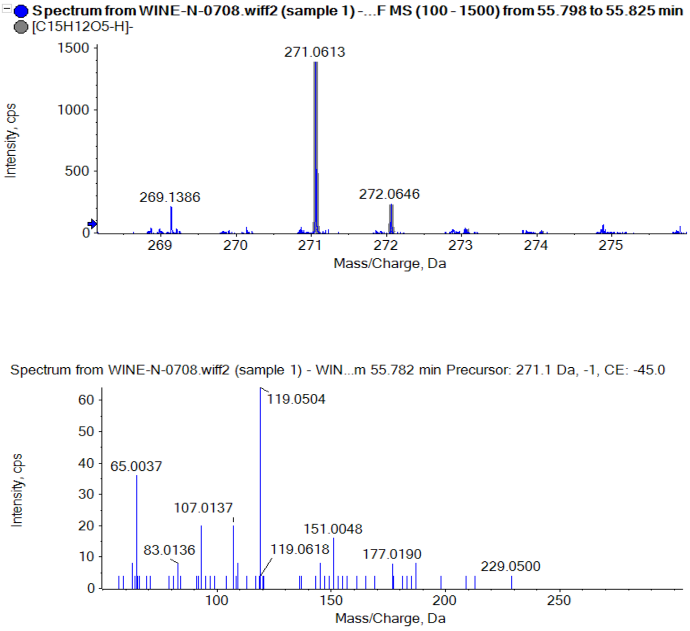

Figure S86. The MS and MS/MS fragments of naringenin.

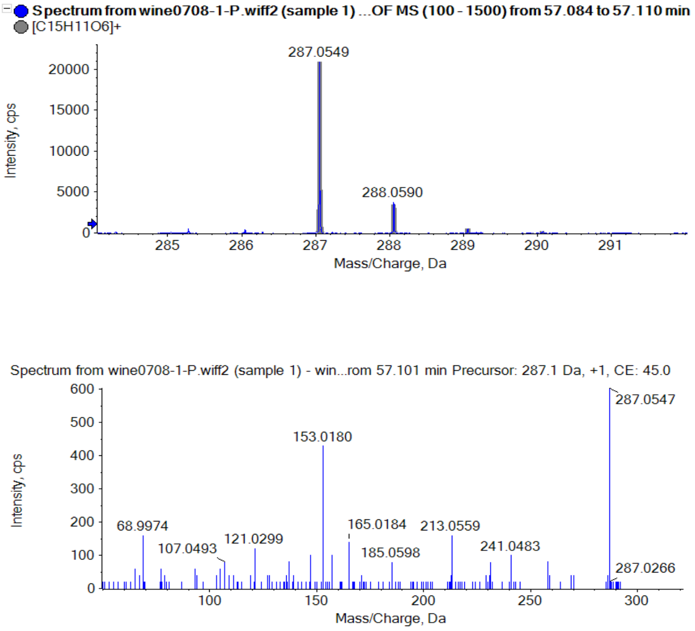

Figure S87. The MS and MS/MS fragments of cyanidin.

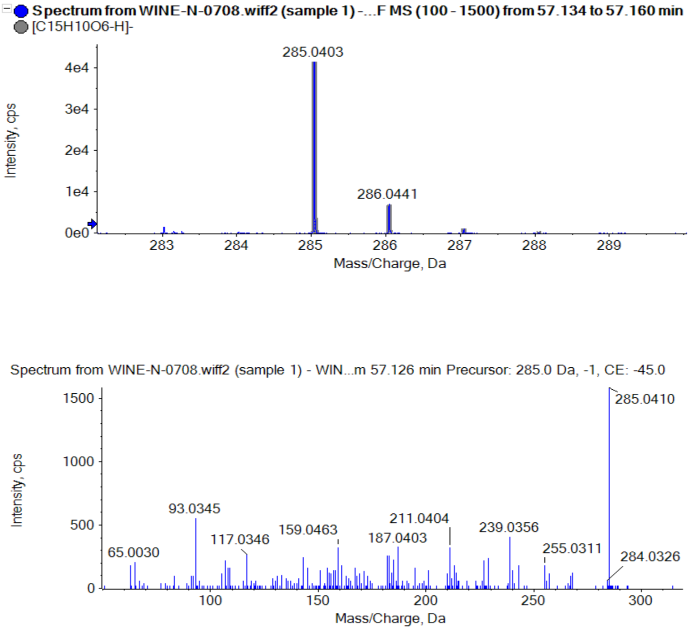

Figure S88. The MS and MS/MS fragments of kamepferol.

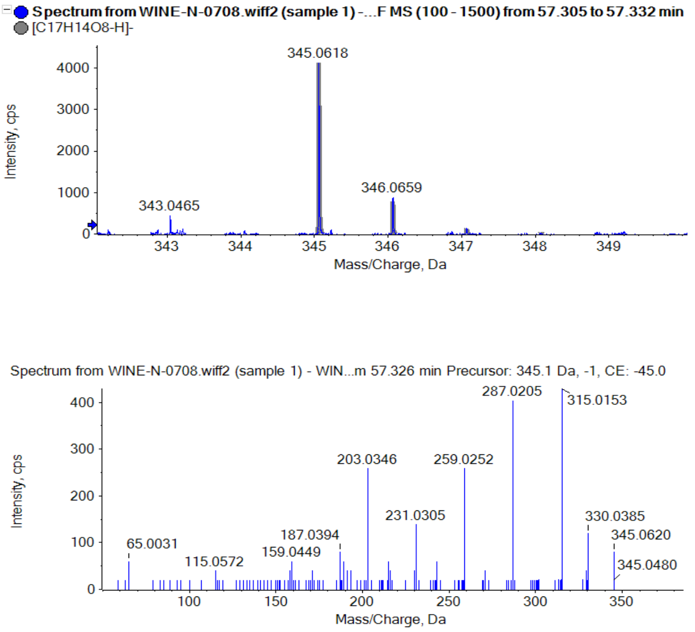

Figure S89. The MS and MS/MS fragments of syringetin.

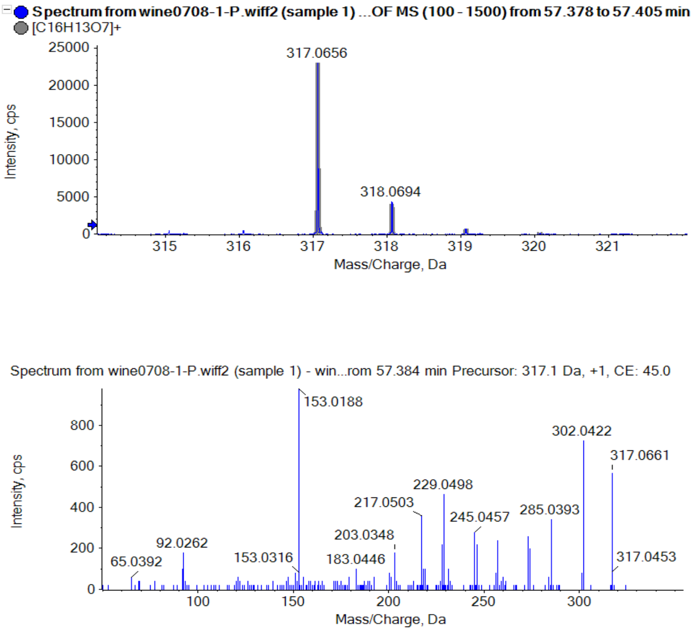

Figure S90. The MS and MS/MS fragments of petunidin.

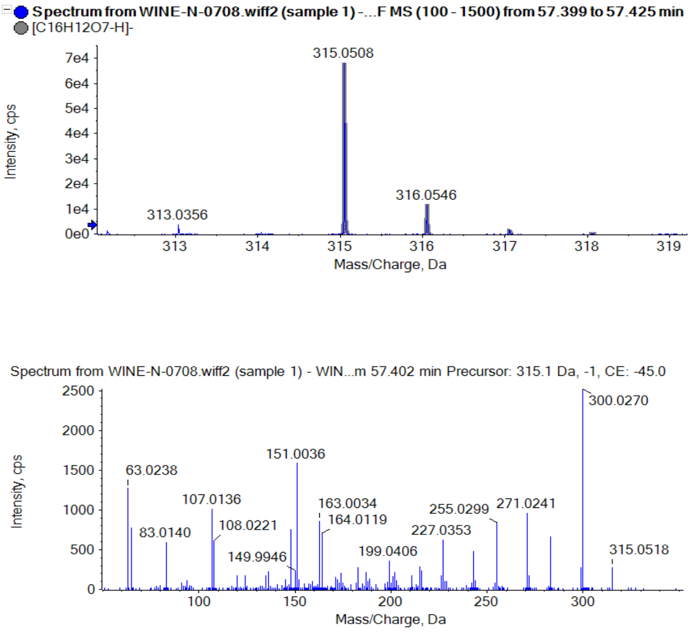

Figure S91. The MS and MS/MS fragments of isorhamnetin.

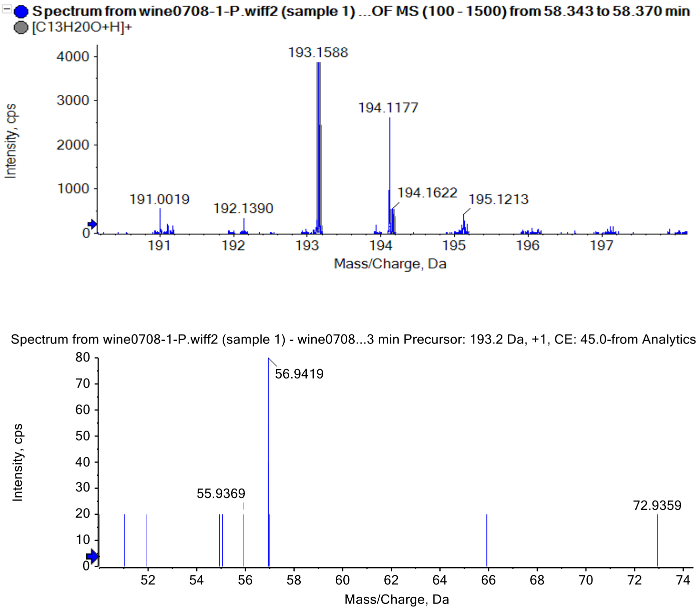

Figure S92. The MS and MS/MS fragments of ionone.

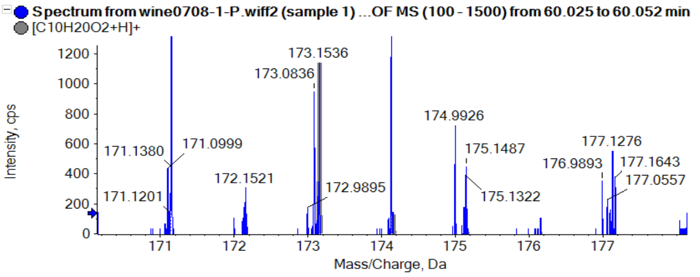

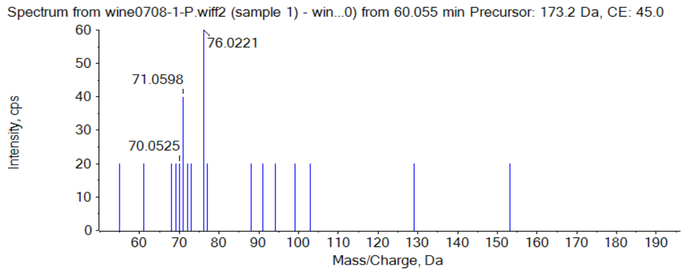

Figure S93. The MS and MS/MS fragments of ethyl caprylate.

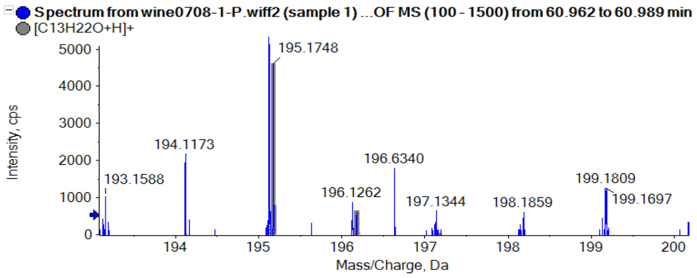

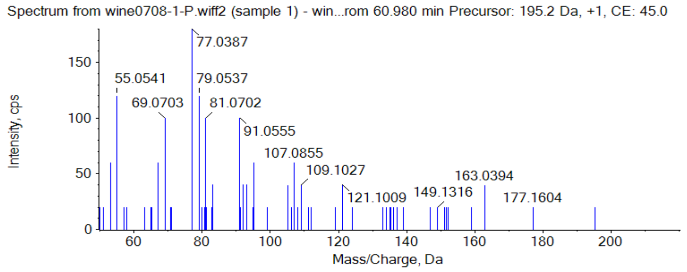

Figure S94. The MS and MS/MS fragments of theaspirane.
